# CRK5 Protein Kinase Contributes to the Progression of Embryogenesis of *Arabidopsis thaliana*

**DOI:** 10.3390/ijms20246120

**Published:** 2019-12-04

**Authors:** Abu Imran Baba, Ildikó Valkai, Nitin M. Labhane, Lilla Koczka, Norbert Andrási, Éva Klement, Zsuzsanna Darula, Katalin F. Medzihradszky, László Szabados, Attila Fehér, Gábor Rigó, Ágnes Cséplő

**Affiliations:** 1 Institute of Plant Biology, Biological Research Centre, 6726 Szeged, Hungary; baba.abuimran@brc.hu (A.I.B.); ildiko.valkai@gmail.com (I.V.); andrasi.norbert@brc.hu (N.A.); szabados.laszlo@brc.hu (L.S.); feher.attila@brc.hu (A.F.); 2 Doctoral School in Biology, Faculty of Science and Informatics, University of Szeged, 6720 Szeged, Hungary; 3 Department of Botany, Bhavan’s College Andheri West, Mumbai 400058, India; nitin.labhane@bhavans.ac.in; 4 Developmental and Cell Biology of Plants, CEITEC Masaryk University, 62500 Brno, Czech Republic; lilla.koczka@ceitec.muni.cz; 5 Department of Plant Biology, University of Szeged, 52. Középfasor, H-6726 Szeged, Hungary; 6 Proteomics Research Group, Biological Research Centre, 6726 Szeged, Hungary; klement.eva@brc.hu (É.K.); darula.zsuzsanna@brc.hu (Z.D.); medzihradszky.katalin@brc.hu (K.F.M.)

**Keywords:** auxin gradient, polar auxin transport (PAT) proteins, GA3, embryogenesis, *Arabidopsis thaliana*, Calcium-Dependent Protein Kinase-Related Kinase (CRK)

## Abstract

The fine tuning of hormone (e.g., auxin and gibberellin) levels and hormone signaling is required for maintaining normal embryogenesis. Embryo polarity, for example, is ensured by the directional movement of auxin that is controlled by various types of auxin transporters. Here, we present pieces of evidence for the auxin-gibberellic acid (GA) hormonal crosstalk during embryo development and the regulatory role of the *Arabidopsis thaliana* Calcium-Dependent Protein Kinase-Related Kinase 5 (AtCRK5) in this regard. It is pointed out that the embryogenesis of the At*crk5-1* mutant is delayed in comparison to the wild type. This delay is accompanied with a decrease in the levels of GA and auxin, as well as the abundance of the polar auxin transport (PAT) proteins PIN1, PIN4, and PIN7 in the mutant embryos. We have previously showed that AtCRK5 can regulate the PIN2 and PIN3 proteins either directly by phosphorylation or indirectly affecting the GA level during the root gravitropic and hypocotyl hook bending responses. In this manuscript, we provide evidence that the AtCRK5 protein kinase can in vitro phosphorylate the hydrophilic loops of additional PIN proteins that are important for embryogenesis. We propose that AtCRK5 can govern embryo development in Arabidopsis through the fine tuning of auxin-GA level and the accumulation of certain polar auxin transport proteins.

## 1. Introduction

Embryogenesis is the first stage of the plant life cycle, which initiates with fertilization and zygote development and it terminates with the maturation of the embryo. The three phases of embryogenesis are the proembryo stage, the early, and the late embryogenesis. Embryo polarity is determined during the proembryo stage with asymmetric division of the zygote [1], while morphogenesis characterizes early embryogenesis from the globular to the heart stage [2]. The late embryogenesis is marked by maturation (expansion of the cotyledons and the axis of the embryo without cell divisions) and ends in dormancy [3]. Maturation also leads to embryo desiccation and the accumulation of storage nutrients in the cotyledons of Arabidopsis [3,4].

Several hormones control plant growth and development. Amongst them, auxin (indole-acetic acid; IAA) is fundamental during the whole plant lifespan [5,6,7,8] and it has a pivotal role in the determination of embryo structure and size at the beginning of seed development [4,9,10,11,12]. Plants have a peculiar way of performing the directional cell-to-cell auxin transport that is required for the generation of a polarized embryonic axis determining the body plan of the adult organism [4]. In Arabidopsis, the PIN-FORMED (PIN) efflux transporters, the members of the AUX1/ LIKE-AUX1 (AUX/LAX) auxin influx protein family, and the ABCB transporter superfamily (PGP proteins), PIN-Like transporters (PILS), and WALLS ARE THIN 1 (WAT1) transport auxin [13,14,15,16,17,18,19,20]. The polar subcellular localization of some of these transporters, especially the PINs, ensures the directional movement of auxin [21]. Amongst the eight PIN proteins found in Arabidopsis, the PIN1, PIN3, PIN4, and PIN7 are expressed during embryogenesis [4,10,18,22,23]. Maternally produced auxin contributes to the earliest phase of embryo development in Arabidopsis [23]. Until the 16-cell stage, PIN7 is located in the apical membranes of the suspensor cells, ensuring auxin transport from the suspensor to the pro-embryo, while PIN1 is localized in the pro-embyo in a non-polarized manner. At the 32-cell-stage, auxin is produced in the apical part of the globular embryo and changing PIN7 polarity (facing to the basal membrane of the suspensor cells) and polarizing PIN1 location to the basal membrane of embryo cells reverses the route of auxin. Auxin accumulates in a PIN1- and PIN4-dependent manner in the hypophysis (the uppermost cell of the suspensor) specifying the root pole [4,10,18]. PIN3 activity was observed during embryogenesis at the basal pole of the heart stage embryo [10]. The PIN4 protein was found to locate at the descendants of the hypophysis and at provascular initials [10]. The important role of the auxin influx carrier AUX1 and LIKE-AUX1 (LAX1) proteins in the formation of embryo shoot and root poles was also confirmed [17,24]. AUX1 was localized in the central cells of a 32-cell-stage embryo, together with LAX2 [17]. Patterning defects in the upper region, as well as in the future root pole, were detected in aux1 lax1 lax2 triple-mutant embryos [17]. Members of the AUX1/LAX family were shown to be redundantly required for correct cell organization in the radicle tip of mature embryos [24]. These data indicate that the auxin efflux and influx carriers collaborate for regulating cell specification, and both types of transporters are important for well-balanced auxin transport that is responsible for embryo polarity determination and normal embryo development [17].

The gibberellins (GAs) are other key hormonal regulators of embryogenesis, in addition to auxin [25,26,27,28,29]. Several data support the crosstalk between gibberellin and auxin, including the regulation of the auxin transport machinery [30,31,32]. It was pointed out that auxin transport is reduced in the inflorescence of the Arabidopsis gibberellin biosynthesis and signaling mutant ga1 [33]. ga1 (ga requiring1; SALK_109115; ecotype Columbia [Col-0]) is a loss-of-function allele that is impaired in an early step of GA biosynthesis, due to an insertion in the gene encoding ENT-COPALYL DIPHOSPHATE SYNTHETASE1 [33,34,35]. The impaired auxin transport in this GA biosynthesis mutant did correlate with the reduction of the abundance of the PIN auxin efflux transporters. Exogenous GA treatment restored the PIN protein levels in the mutant to those of wild type. The experiments indicated that PIN2 was targeted for vacuolar degradation as a consequence of GA deficiency, leading to a reduction in the auxin transport. The impairment of embryo cotyledon differentiation and root gravitropic response, two PIN-dependent phenotypes of GA biosynthesis and signaling mutants of Arabidopsis, were also correlated with reduced auxin transport [33].

The essential role of GAs in late embryogenesis was recently pointed out by [3]. Some GA biosynthesis genes are activated at the bent cotyledon embryo stage, which is followed by the activation of proteolytic enzymes and α- amylases [36]. GAs and ABA have closely correlated, but antagonistic actions on seed development [4,37,38,39]. The processes of seed maturation (accumulation of nutrients in the endosperm/embryo) and desiccation (embryo dormancy stage to survive hydric stress) are mainly regulated by ABA. The concentration of ABA elevates during seed maturation and desiccation, whilst the active GA concentration decreases, as reviewed by [4]. The process of imbibition—recovery of the desiccated seed from the water deficit to mobilize enzymes and to break dormancy—is regulated by GA. GA triggers germination by mobilizing the embryo resources [40]. Therefore, the changing ratio of ABA and GAs regulates the processes of seed maturation and germination [4]. The transcription factors ABSCISIC ACID INSENSITIVE 3 (ABI3), FUSCA3 (FUS3), and LEAFY COTYLEDON 1 and 2 (LEC1 and LEC2), called together as the AFL factors, are considered to be master regulators of ABA-dependent seed maturation processes and the late embryogenesis stage in Arabidopsis [3,41,42,43,44,45,46]. Among others, they control the accumulation of storage compounds, the acquisition of desiccation tolerance and dormancy [46], and play a role in the repression of post-germination processes during embryogenesis [47].

Auxin is implicated not only in early embryogenesis, but also in late seed development. The auxin biosynthesis YUCCA gene family members (*YUC1, YUC4, YUC10, YUC11*) also exhibit functional redundancy during early and late embryogenesis [48]. Auxin controls seed dormancy in Arabidopsis in cross-talk with abscissic acid (ABA) signaling [4,49]. Moreover, the high auxin concentration that is characteristic for seed development promotes the accumulation of active GAs by regulating the expression of several GA metabolism genes, like gibberellin 20-oxidase (*AtGA20ox*) or gibberellin 2-oxidase (*AtGA2ox*) [50,51,52].

In Arabidopsis, two enzymes, GA20ox and gibberellin 3-oxidase (GA3ox), catalyze the conversion of gibberellin intermediates to their bioactive forms, while GA2ox catabolizes bioactive gibberellins [28,29]. The GID1 receptor perceives the activated gibberellins, which triggers the degradation of DELLA proteins, which are the key repressors of gibberellin signaling [3]. Arabidopsis has five DELLA proteins, namely GA-INSENSITIVE (GAI), REPRESSOR OF ga1-3 (RGA), RGA-LIKE 1 (RGL1), RGL2, and RGL3, and these accomplish partially redundant and disparate functions in gibberellin-regulated developmental processes [53,54,55]. In the GA signaling pathway, the DELLAs perform their canonical transcriptional regulation function mainly through physical interaction with transcription factors and other regulatory proteins [3,56] or participate in a non-transcriptional branch of GA signaling via recycling proteins to the plasma membrane instead of their vacuolar degradation [32]. The DELLAs interact with the LEAFY COTYLEDON1 (LEC1) protein during late embryo development [3]. At this specific period, the bioactive gibberellins are biosynthesized in large quantities, triggering the degradation of DELLAs for achieving the transcriptional activity of LEC1, thus finally promoting embryo development [3,4].

The AtCRK5 protein kinase is active in most Arabidopsis organs and it is involved in the establishment of the proper auxin gradient that is necessary for the gravitropic response of the root and the bending of the hypocotyl during skotomorphogenesis [57,58,59]. Here, we describe the importance of the CRK5 protein kinase in Arabidopsis embryo development. We found that the progression of embryogenesis was delayed in the At*crk5-1* mutant having reduced gibberellic acid (GA) content and shorter SAM-RAM distance in late embryo stages. The shifted embryo developmental stages and smaller embryo size could be restored in the mutant embryos by exogenous GA treatment. Previously, we showed that the CRK5 kinase is able to phosphorylate the PIN2 [57,58] and PIN3 [59] auxin transporters and its mutation results in impaired auxin transport. Therefore, we tested whether this protein kinase also regulates the auxin flow during embryogenesis and if the delayed embryogenesis of the mutant can be ascribed to altered auxin transport efficiency. We could observe decreased levels of auxin at the different developmental stages of At*crk5-1* mutant embryos while using the DR5::GFP auxin reporter. Moreover, detecting GFP-tagged proteins, the decreased abundance of the polar auxin transport proteins PIN1, PIN4, PIN7, and AUX1 could also be observed in all embryo stages. Our findings suggest that—in addition to its regulatory role in root gravitropic and hypocotyl hook bending responses—AtCRK5 also governs Arabidopsis embryo development by fine tuning the auxin-GA homeostasis and potentially by phosphorylating various polar auxin transport (PAT) proteins.

## 2. Results

### 2.1. The Atcrk5-1 Mutant Exhibits a Considerable Delay in the Progression of the Phases of Embryogenesis

**Figure 1:**
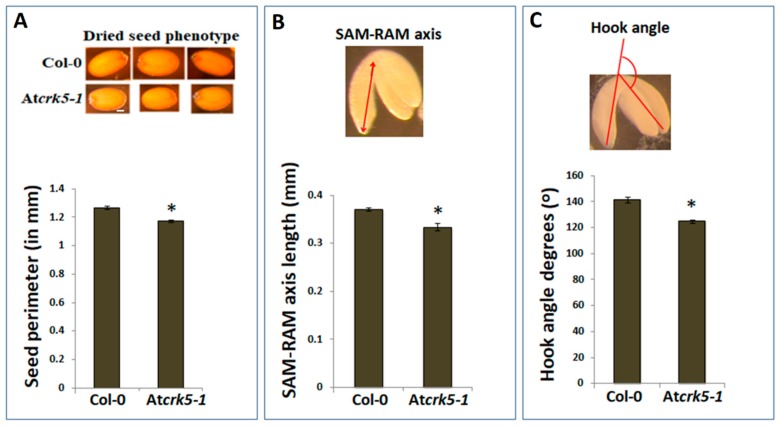
Differences in seed and embryo sizes and embryo bent-cotyledon hook angles of wild type (Col-0) and mutant (At*crk5-1)* lines. (**A**) Pictures show dry seeds of the wild type (Col-0) and mutant (At*crk5-1).* The diagram shows the quantification of the seed size in wild type and mutant lines after two days’ imbibition of dried seeds at 4 °C. The seed contours were measured by ImageJ. The averages are from 100 independent seeds from the wild type and mutant, respectively. Standard errors (SE) are also shown. The mutant value indicated by asterisk is significantly lower compared to the wild type (Student’s t-test: *p* < 0.01, *n* = 100). (**B**) Embryo axis length measured from shoot apical meristem (SAM) until root apical meristem (RAM) was calculated in bent-cotyledon-stage embryos arised from the (**A**) experiment. Pictogram shows how the SAM-RAM axis was measured. All values are averages of at least 100 bent-cotyledon embryos. Standard errors (SE) are also shown. Asterisk depicts significant difference between the wild type and mutant embryos (Student’s t-test: *p* < 0.05, *n* = 100). (**C**) Differences in the hook angles of wild type and mutant embryos at the bent-cotyledon-stage (after two days’ imbibition of seeds at 4 °C). The averages and standard errors (SE) are shown. The mutant value is significantly different in comparison with the wild type (Student’s t-test: * *p* < 0.01, *n* = 75). Pictogram shows the mode of measuring embryo hook angles (**C**). All experiment was repeated three times.

We could recognize a notable size difference when comparing the wild type *Arabidopsis thaliana* (Col-0) and the At*crk5-1* mutant dry seeds (containing bent-cotyledon embryo stages) (Figure 1A, pictures). We could determine significantly smaller seed sizes for the mutant when the dried seed contours were measured (Figure 1A, diagram). After two days’ imbibition of dried seeds at 4 °C, we carefully pressed out the inverted stage embryos from the seeds by forceps, and measured the average length of the embryo axis from the shoot apical meristem (SAM) to the root apical meristem (RAM) in both of the genotypes. We found that the length of the axis was significantly shorter in At*crk5-1* embryos as compared to the wild type Col-0 ones (Figure 1B). Additionally, the bending angles of hypocotyl hooks of these bent-cotyledon-stage embryos were also measured. It is notable that the hypocotyl hook of the At*crk5-1* mutant embryos was already more opened at this stage when compared to the wild type ones (Figure 1C; see Reference 61. for more details).

**Figure 2:**
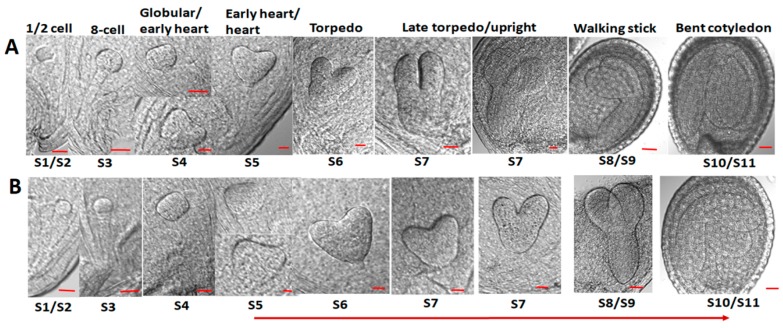
Embryogenesis of the wild type *Arabidopsis thaliana* Col-0 and the At*crk5-1* mutant. Bright field microscopic images from a CELL-R Olympus Microscope. Embryo shapes were visualized after chloral hydrate treatment. S1–S11 = silique developmental stages where S1 represents the youngest and S11 the oldest siliques formed after pollination. As compared to wild type (**A**) embryos, there is a shift in At*crk5-1* mutant (**B**) embryo development initiated from the globular/early heart embryo stages found in silique5 (S5). Red arrow represents the start and sequential direction of delay. Note that the wild type (Col-0) bent-cotyledon-stage embryos have 180° hook bending, while the mutant embryos at the same developmental stage (At*crk5-1*) have much less (around 130°) bending angle. (Scale bars = 10 µm for S1–S7 and 100 µm for S9–S11).

Thereafter, the individual developmental stages of the embryos were tested to find possible morphological differences between the wild type and mutant embryos. Microscopic images of embryos photographed by a CELL-R Olympus Microscope, are presented in Figure 2. The embryos were isolated from siliques1–11 (where S1–S11 represent silique position numbers from the youngest to oldest) of greenhouse plants of the same age. A sequential shift could be observed in the stages of At*crk5-1* embryo development from the globular/early heart embryo stages onward (from silique4; S4) when compared to the wild type embryos (Figure 2). Moreover, in the S10–S11 siliques, the At*crk5-1* embryos considerably differed in morphology from the wild type ones (Figure 2).

**Figure 3:**
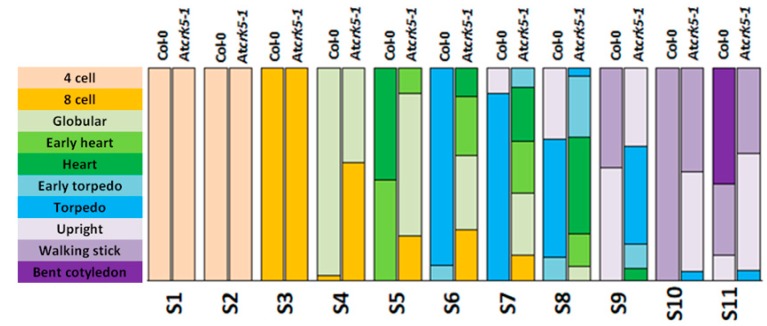
Quantitative analysis of embryogenesis in wild type *Arabidopsis thaliana* (Col-0) and At*crk5-1* mutant in correlation to silique development. Distribution of the different embryo developmental stages from S1 until S11 depicted in percentage (%). A notable shift in At*crk5-1* embryo development can be observed from the globular embryo stages in silique 4 (S4). Minimum 50 seeds were investigated for both genotypes for each embryo developmental stage of green-house-grown plants. The experiments were repeated three times with the same results.

Figure 3 indicates quantitative analysis of the progression of embryo development in wild type and At*crk5-1* siliques. The distribution of sequential embryo developmental phases in the S1–S11 siliques is indicated in percentage by color coding the different stages. These quantitative data indicate that, in the wild type, the S4/S5 siliques contained basically globular, early heart and heart embryos, while in the same type of siliques of the mutant mainly had the eight-cell, globular, and, at a lesser extent, early heart embryos. The wild type S6 siliques mainly consisted of torpedo and early torpedo embryos, while the mutant S6 siliques were dominated by heart-stage embryos but still also contained eight-cell and globular type embryos. The S7 siliques of the wild type had mostly late torpedo and, at a lesser extent, upright embryos, while the mutant S7 siliques still mainly had embryos in the globular and heart (and a few percentage of early torpedo) embryo stages. In the S8/S9 siliques of the wild type, there were mainly torpedo, upright, and walking stick (app. 90° cotyledon bending) stage embryos. In contrast, the mutant S8/S9 siliques had still embryos in the early heart, heart, early torpedo, and torpedo stages with a few percentage of upright ones. The wild type S10/S11 siliques already contained principally walking stick and bent-cotyledon embryos, but the S10/S11 siliques from the mutant only contained upright and walking stick embryos (Figure 3).

**Figure 4:**
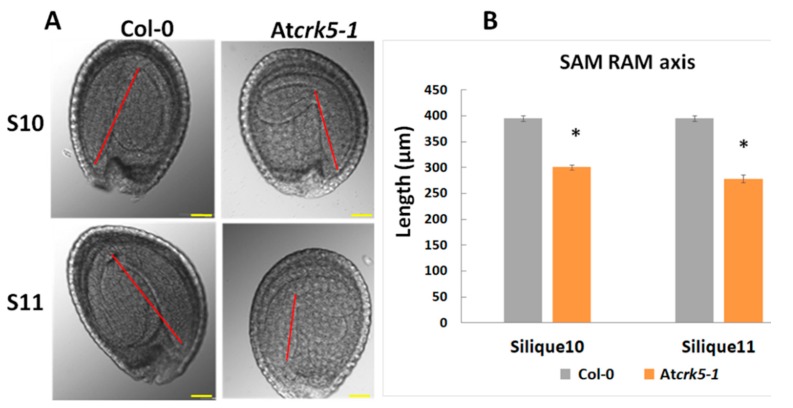
Comparison of hypocotyl bending and the SAM-RAM distance of bent-cotyledon-stage embryos. (**A**) Embryo length was measured from SAM to RAM in silique10 and silique11-derived embryos after preclearing in Hoyer’s solution. The SAM-RAM axis is indicated with red line. Note that the axes of the At*crk5-1* embryos are shorter in comparison to those of the wild type. (**B**) Quantification of embryo axis length differences. Asterisks depict significant differences between the wild type and mutant embryos (Student’s t-test: * *p* < 0.05, *n* ≥ 50). Scale bars = 100 μm.

Therefore, embryos in the S11 siliques of the At*crk5-1* mutant resembled those in the S9 siliques of the wild type, which supported that, at the end of the investigations, the embryo development was delayed approx. by two phases in the mutant as compared to the wild type Col-0 (Figure 2 and Figure 3). Furthermore, the hypocotyl hook of the embryos in the bent-cotyledon-stage of S10/S11 siliques was considerably less closed in the mutant than in the wild type (Figure 4A). We found significant decrease in the axis length when we compared the SAM-RAM distance of the mutant embryos to that of the wild type Col-0 in the S10–S11 siliques, (Figure 4B).

**Figure 5:**
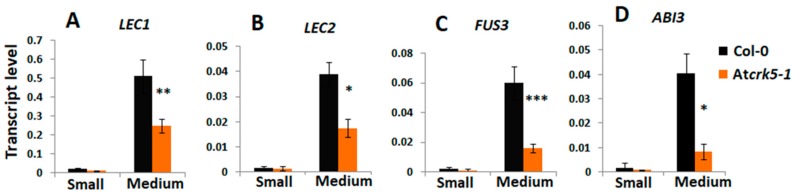
Expression level of embryo maturation/dormancy related genes at different silique stages quantified by qRT-PCR. The relative transcript levels were determined in small siliques (S; S1–S4, 2DAP) and medium siliques (M, S5–S7, 3DAP) for (**A**) *LEC1*, (**B**) *LEC2*, (**C**) *FUS3*, and (**D**) *ABI3*. *Ubiquitin1 (UBQ-1)* was used as an internal control. The values are means (+/−) SD of two independent biological repeats. Student’s t-test: * *p* < 0.05, ** *p* < 0.01, *** *p* < 0.001. Primers used are listed in Table S1.

The relative expression of genes coding for the transcription factors LEC1, LEC2, FUS3, and ABI3 involved in embryo maturation and dormancy regulation processes were investigated to further support the delay in embryo development. Small (S1-S4) and medium-sized (S5-S7) siliques of the wild type (Col-0) and mutant (Atcrk5-1) plants were collected and analysed by qRT-PCR. The transcription factors LEC1, LEC2, and FUS3 act at late embryogenesis; therefore, we did not notice gene expression in small siliques. In the medium size siliques (from S5–S7), these genes were significantly less expressed in the *Atcrk5-1* mutant than in the control (Figure 5A–C). The expression level of the *ABI3* transcription factor gene was also dropped at each silique stage in the *Atcrk5-1* mutant (Figure 5D). The reduced transcription factor expressions in medium-sized siliques are in accordance with the delayed embryogenesis in the seeds of the At*crk5-1* mutant.

### 2.2. The Delayed Development of the Atcrk5-1 Embryos Is Linked to Their Decreased Gibberellin Synthesis and Level

**Figure 6:**
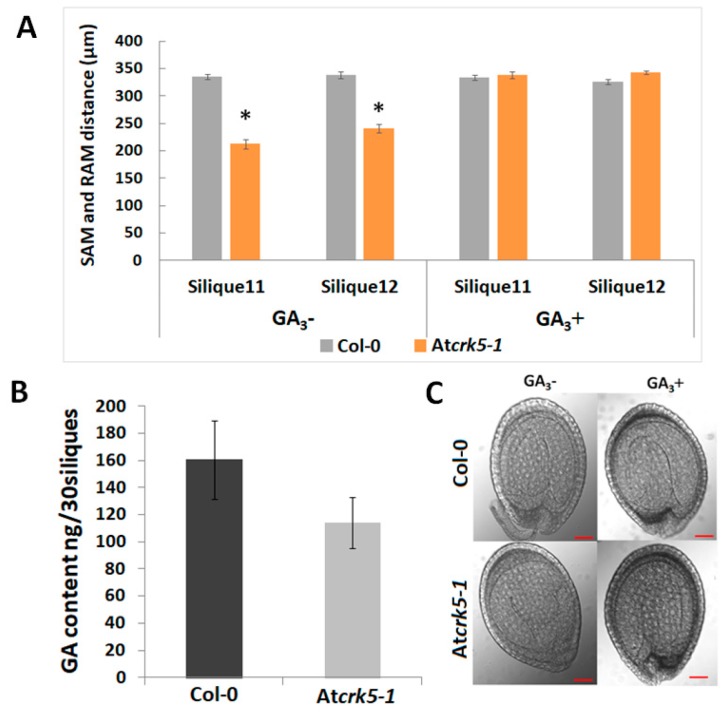
Role of gibberellic acid in the delayed embryo development of At*crk5-1*. (**A**) Quantification of embryo axis length differences in siliques S11–S12. Embryo axis from SAM-to-RAM was measured after the treatment of developing inflorescences of wild type (Col-0) and At*crk5-1* mutant plants without (GA −) or with 20 μM GA_3_ (GA +). The exogenous GA_3_ supply restored the length of the mutant embryos to the wild type level. (Student’s t-test:* *p* < 0.05, *n* = 30). The data presented are the means of two biological repeats. (**B**) Quantification of the total GA content in medium stage siliques (S5–S6–S7). 30-30 siliques were collected from wild type (Col-0) and mutant (At*crk5-1*) inflorescence. The total GA content was determined using a GA-antibody-based Elisa kit. (**C**) Rescue of the delayed embryo bending phenotype in S11 of the At*crk5-1* mutant by 20 μM GA_3_ treatment with its noticeable change from walking stick to bent cotyledon stage, while Col-0 remains in the bent cotyledon stage. Representative images are shown from experiments repeated two times with 30-30 mutant and wild type embryos. Scale bars = 100 µm.

The delay in At*crk5-1* embryo development started at the globular/early heart embryo stages found in silique4 (S4). The rapid expansion of wild type embryos takes place in this period (from S5 till S11) [3]. This was correlated with the elevated levels of bioactive gibberellins GA_1_ and GA_3_ reaching their maximum at the walking stick embryo stage [3]. Bioactive gibberellins regulate late embryogenesis via modulating DELLA protein level in the embryos. GA deficiency and DELLA protein accumulation both led to abnormal embryos that are characterized by a shortened embryo axis and unbended cotyledons [3]. Based on these observations, the delayed morphogenesis of the At*crk5-1* embryos might be explained by the reduced level of bioactive GAs. Therefore, a GA-antibody-based Elisa kit measured the total GA content of medium-sized siliques (S5–to–S7) collected from mutant and wild type plants. It was found that the medium-sized siliques of the At*crk5-1* mutant contained less GA than those of the wild type plants at the same age (Figure 6A). We found that GA_3_ treatment restored the SAM-RAM distance of the mutant embryos to the wild type level at S11–S12 stages when we treated greenhouse-grown wild type Arabidopsis and At*crk5-1* mutant plants by 20 µM GA_3_ from the early development of the inflorescence onward (Figure 6B). Additionally, the hypocotyl bending phenotype of the At*crk5-1* mutant embryos found in S10–S12 (Figure 4) was also restored to the wild type level by the exogenous GA_3_ treatment (Figure 6C).

**Figure 7:**
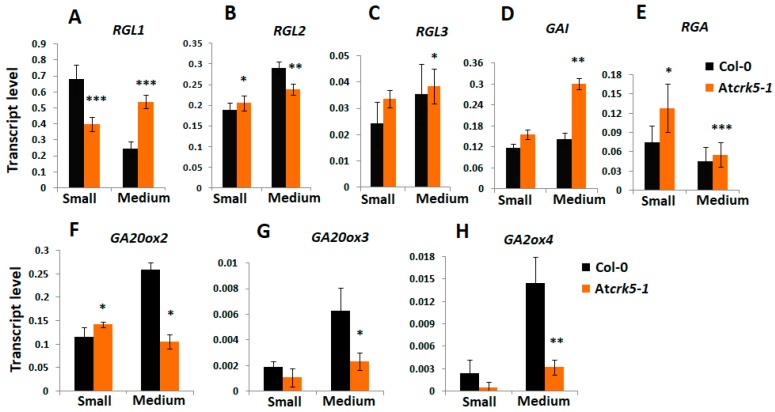
Relative expression level of selected genes involved in Gibberellic Acid (GA) metabolism and signaling at different silique stages quantified by qRT-PCR. The relative transcript levels were determined in small siliques (S; S1–S4, 3DAP) and medium siliques (M, S5–S7, 5DAP) for the DELLA GA signaling genes (**A**) *RGL1*, (**B**) *RGL2*, (**C**) *RGL3*, (**D**) *GA-INSENSITIVE* (*GAI*), and (**E**) *REPRESSOR OF ga1-3* (*RGA)*, the gibberellin biosynthesis genes (**F**) *GA20ox2* and (**G***) GA20ox3*, and the gene of the GA catabolism enzyme (**H***) GA2ox4*. *Ubiquitin1* (*UBQ-1*) was used as an internal control. The values are means (+/−) SD of two independent biological repeats. Student’s t-test: * *p* < 0.05, ** *p* < 0.01, *** *p* < 0.001. Primers used are listed in the Table S1.

We also examined the gene expression levels of the gibberellin 20-oxidases (*AtGA20ox2* and *AtGA20ox3*), which are highly expressed GA biosynthesis enzymes in dry and imbibed seeds [51] and they are responsible for converting the inactive intermediates of GA biosynthesis into bioactive forms [3]. The *AtGA20ox2* and *AtGA20ox3* genes have high expression during the expansion stage of embryogenesis (4–7 days after pollination, 4–7 DAP) [3]. The expression level of the gibberellin deactivation gene *GA2ox4*, which also has a high expression level in embryos at this stage (4–7 DAP; [3]), was also tested. Furthermore, the genes of DELLA proteins canonically known to participate in GA signaling during embryogenesis [3,32] were included into the gene expression study. Siliques of wild type (Col-0) and mutant (At*crk5-1*) plants were collected according to their developmental stages, like small (three DAP) and medium (five DAP) ones corresponding to embryo developmental stages S1–S4 and S5–S6–S7, respectively. The gene expression analysis was focused on the medium silique stage, where decreased GA content was observed in the At*crk5-1* mutant. When considering the genes coding for DELLA proteins, we found a slight gene expression level elevation in *RGL1* (Figure 7A) and *GAI* (Figure 7D) genes, while the expression levels of the *RGL2*, *RGL3*, and *RGA* (Figure 7B,C,E) genes did not change in the *Atcrk5-1* as compared to that of the wild type. However, the *GA20ox2/GA20ox3* gene expression levels were decreased in the siliques of the At*crk5-1* mutant in comparison to the wild type (Figure 7F,G). This indicates that the insufficient conversion of the inactive GA intermediates into their bioactive forms might cause the fall-off in the GA level at the medium silique stage (Figure 6B). The expression level of the *GA2ox4* was also decreased (Figure 7H) in the At*crk5-1* medium siliques, which might relate to the reduced negative feedback of GAs on their biosynthesis.

### 2.3. The Auxin Level Is Decreased in the Atcrk5-1 Mutant Embryos in Comparison with the Wild Type Ones.

**Figure 8:**
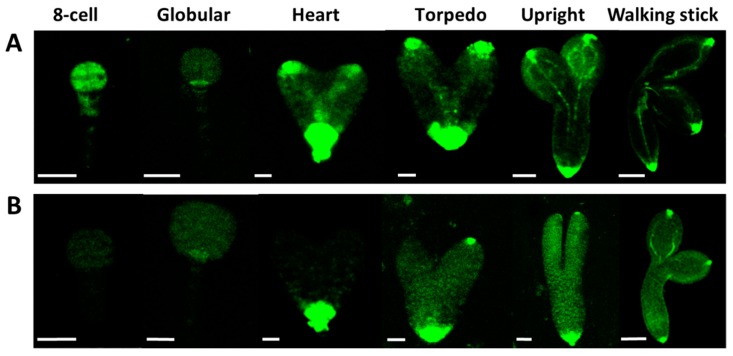
The auxin sensor DR5::GFP activity was recorded in wild type *Arabidopsis* and mutant At*crk5-1* embryos at several developmental stages. Auxin distribution during the embryo development in wild type (**A**) and in the mutant (**B**). In both cases, 5-5 embryos were investigated from three independent experiments. Note the decreased auxin levels in each embryo developmental stage in the At*crk5-1* mutant. Scale bars: eight-cell to torpedo = 15 µm; upright and walking stick = 100 µm.

It is known that there is crosstalk between auxin and GA during plant developmental regulation, including embryogenesis [52,60]. We pointed out a GA shortage in the At*crk5-1* embryos at the globular/ heart/torpedo embryo stages (in medium siliques S5–S7, Figure 6B). Among others, GA is required for proper auxin transport in Arabidopsis [33]. Therefore, we tested the auxin distribution in the At*crk5-1* mutant embryos during their development. The DR5::GFP marker (a synthetic promoter that responds to the presence of auxin linked to GFP gene [61]) was introduced into wild type (Figure 8A) and At*crk5-1* plants (Figure 8B). Fluorescence microscopy was used to detect auxin-regulated GFP fluorescence in various embryo stages. We found a strong decrease in the DR5::GFP signal in the At*crk5-1* embryos all along from the eight-cell stage until the walking stick stage of the embryos (Figure 8).

### 2.4. Expression and Abundance of Auxin Efflux Proteins During Embryogenesis of Wild Type and Atcrk5-1 Mutant Plants

**Figure 9:**
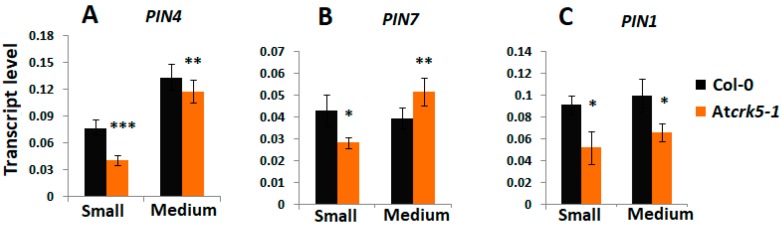
Relative expression level of auxin efflux carrier *PIN* genes at different silique stages quantified by qRT-PCR. The relative transcript levels were determined in small siliques (S1–S4, 3DAP) and medium siliques (S5–S7, 5DAP) for (**A**) *PIN4*, (**B**) *PIN7*, (**C**) *PIN1*. *Ubiquitin1 (UBQ-1)* was used as an internal control. The values are means (+/−) SD of two independent biological repeats. Student’s t-test: * *p* < 0.05, ** *p* < 0.01, *** *p* < 0.001. Primers used are listed in the Table S1.

Auxin is known to determine cell division patterns and cell specification during all stages of embryogenesis, starting from the first division of the zygote and this is strongly dependent on PINs localization and activity [4,10,18,60]. The expression and abundance of the auxin efflux transporters PIN1, PIN3, PIN4, and PIN7 were investigated at various embryo developmental stages in order to investigate whether the delayed embryogenesis of the At*crk5-1* mutant is associated with altered auxin transport pattern or efficiency. The *PIN4* and *PIN7* gene expressions were reduced in the small silique stage (S1–S4) of the *Atcrk5-1* mutant (Figure 9A,B), while the *PIN1* gene expression was down regulated in the mutant siliques during the small and medium stages inclusive the torpedo stage (S1–S4 and S5–S7, Figure 9C).

GFP-tagged PIN proteins were produced in wild type (Col-0) and mutant (At*crk5-1*) backgrounds to investigate their protein level and distribution. At early embryogenesis, PIN1 and PIN7 are required for normal embryo development. In the proembryo, PIN1 first localizes non-polarly but, at the globular stage, it becomes localized to the basal membrane of provascular cells facing the hypophysis [10]. During early embryogenesis, PIN7 first localizes at the apical side of suspensor cells and at the 32-cell (globular embryo) stage PIN7 polarity changes and the protein becomes associated with the basal membrane of suspensor cells. PIN1 and PIN7 polarization changes correlate with the reversion of the auxin flow [4,10,12].

**Figure 10:**
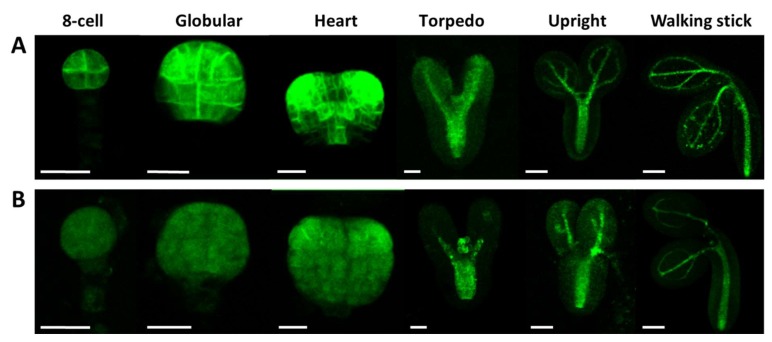
Level and distribution of PIN1-GFP in wild type (**A**) and in At*crk5-1* mutant (**B**) embryos during embryogenesis. Note that the PIN1-GFP signal is much less intense in the At*crk5-1* mutant then in the control embryos. In both cases 5-5 embryos were investigated from three independent experiments. Scale bars: eight-cell to torpedo = 15 µm; upright; and, walking stick = 100 µm.

**Figure 11:**
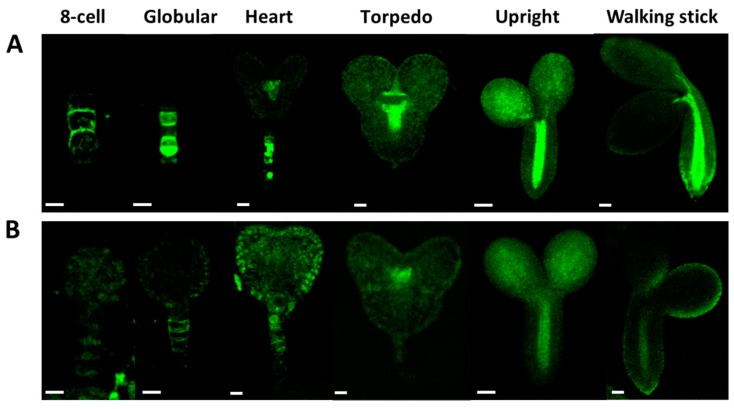
Comparison of the PIN7-GFP abundance and distribution in developing Arabidopsis embryos. PIN7-GFP intensity distribution during embryo development in wild type (**A**) and in the mutant (**B**). Note the decreased abundance of PIN7-GFP in each developmental stage of At*crk5-1* embryos. In both cases 5-5 embryos were investigated from three independent experiments. Scale bars: eight-cell to torpedo = 15 µm; upright/walking stick= 100 µm.

The pattern of PIN1/PIN7 distribution seems to be unaffected by the At*crk5-1* mutation (Figure 10 and Figure 11). Figure 10 shows PIN1-GFP abundance in wild type (A) and At*crk5-1* (B) embryos from the eight-cell stage to the walking stick embryo stage. The PIN1-GFP signal intensity is lower in the At*crk5-1* mutant compared to the wild type in all embryogenesis stages, but especially in the early ones (up to the heart stage). Likewise, the PIN7-GFP signal intensity was drastically decreased in the At*crk5-1* embryos at each embryo stage (Figure 11).

The abundance and distribution of the PIN3 and PIN4 proteins was also investigated. PIN3 is expressed at the basal pole of heart stage embryos during embryo development [10]. For PIN3-GFP, we did not find any abundance or localization alterations in the At*crk5-1* mutant when compared to the wild type during embryo development (Figure S1). The PIN4 protein first appears during early embryogenesis at the globular stage in the descendants of the hypophysial cell and in the provascular initials of root meristem [10]. We have found that the PIN4-GFP signal intensity was also decreased in the At*crk5-1* embryos in comparison to the wild type, like in the cases of PIN1-GFP and PIN7-GFP (Figure S2).

### 2.5. Expression and Abundance of Auxin Influx (AUX1) Proteins During Embryogenesis of Wild Type and Atcrk5-1 Mutant Plants

**Figure 12:**
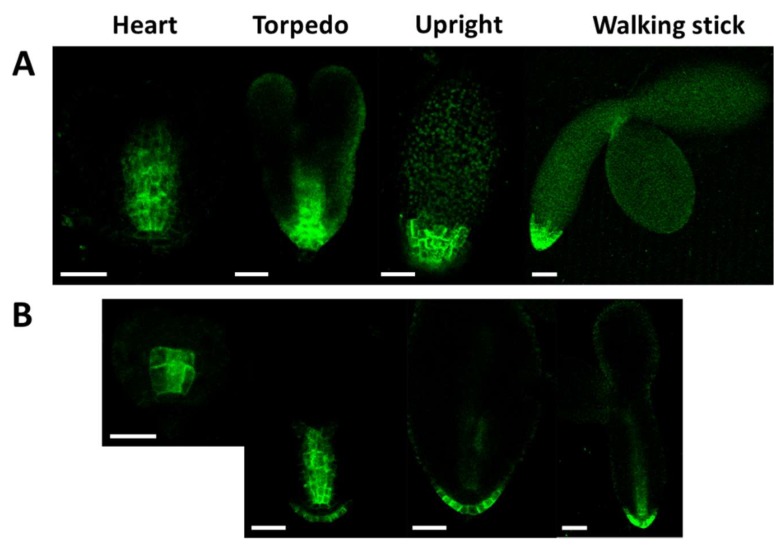
Distribution of AUX1::YFP during embryo development in the wild type (**A**) and in the At*crk5-1* mutant (**B**)**.** In all embryo developmental stages there is lower level of AUX1-YFP in the At*crk5-1* mutant. The localisation of the auxin influx protein (AUX-YFP) is also different in the mutant and the wild type backgrounds. In both cases 5-5 embryos were investigated from three independent experiments. Scale bars = 100 μm.

Auxin influx is also required for proper embryogenesis. AUX1, LAX1, and LAX2 are required for both shoot and root pole formation of embryos, in cooperation with PIN efflux carriers [17,24]. AUX/LAX protein expressions start from the heart stage in Arabidopsis embryos. We compared the localization pattern of the AUX1::YFP signal in the wild type and mutant (At*crk5-1*) embryos during different phases of embryo development (Figure 12). We found that the AUX1::YFP expression level was decreased in the At*crk5-1* mutant in all of the embryo developmental stages in comparison to the wild type. Moreover, the distribution of the AUX1::YFP protein was more restricted at the root pole in the mutant background.

### 2.6. AtCRK5 Can Phosphorylate the Auxin Efflux Proteins PIN1, PIN4 and PIN7 in vitro

**Figure 13:**
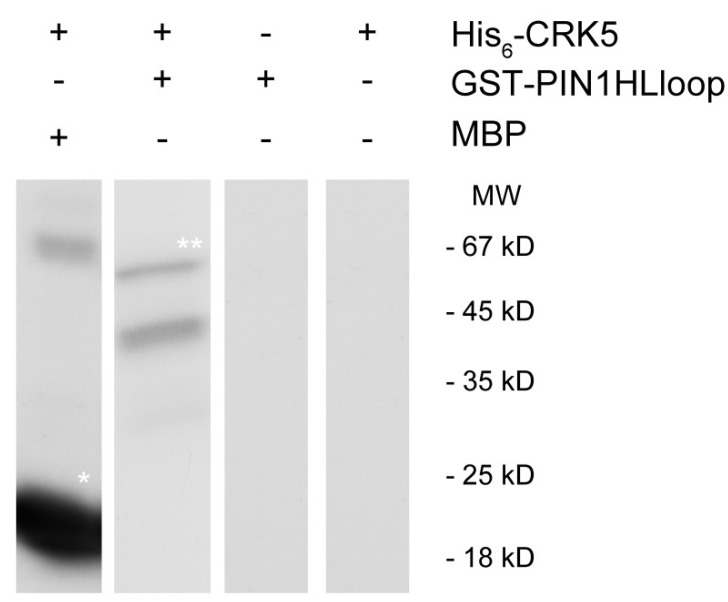
AtCRK5 phosphorylates the PIN1 hydrophilic loop *in vitro*. We performed in vitro radioactive phosphorylation assay with His6-AtCRK5 and two substrates: Myelin Basic Protein (MBP) as a positive control, and GST-PIN1-HL loop. White asterisks indicate the phosphorylation event on MBP (* asterisk) and GST-PIN1HLloop (** asterisk) proteins, respectively. We carried out glutathione S-Transferase (GST) column purification after the kinase reactions shown in the last three columns to remove the HIS6-CRK5 kinase because the His6-CRK5 and the GST-PIN1 protein sizes are nearly identical and we could not distinguish the phosphorylation signals.

The auxin efflux PIN1, PIN3, PIN4, and PIN7 proteins are all supposed to participate in auxin transportation during embryogenesis [17,20,60,62]. The localization and activation of the various PIN proteins are known to be affected by their phosphorylation status [15,63,64,65]. We have already published that AtCRK5 phosphorylates the PIN2 and PIN3 auxin efflux transporters and this may have a role in the gravitropic response of the *Arabidopsis* root upon gravistimulation and the hypocotyl hook bending during skotomorphogenesis [57,59]. The decreased abundance of PIN1-GFP, PIN4-GFP, and PIN7-GFP in At*crk5-1* mutant embryos might reflect a defect in PIN protein stability and/or localization. We speculated that all PIN proteins might be targets of phosphorylation by the AtCRK5 kinase based on the high sequence homology between different PIN hydrophilic loop regions. We cloned the hydrophilic loop domain regions of PIN1, PIN4, and PIN7 into a bacterial protein expression vector in order to support this hypothesis. After transformation and expression into *Escherichia coli* cells, we purified the GST (Glutathione S-Transferase) tagged GST-PIN1HL and MBP (Maltose Binding Protein) tagged MBP-PIN4HL and MBP-PIN7HL. In the case of GST-PIN1HL, we performed an in vitro radioactive kinase assay while using the purified AtCRK5 protein kinase. Figure 13 shows the result of this phosphorylation assay.

Furthermore, a non-radioactive kinase assay was performed to determine whether the AtCRK5 kinase could phosphorylate the PIN4HL and/or the PIN7HL loop regions. The proteins of the kinase reaction were separated in a denaturing polyacrylamide gel and the GST-PIN1HL, MBP-PIN4HL, and MBP-PIN7HL protein bands were excised and sent for analysis by mass spectrometry after staining with Coomassie dye.

We could identify some potential phosphorylation sites for the PIN1, PIN4, and PIN7 hydrophilic loop. AtCRK5 protein kinase can phosphorylate the PIN1 protein at Ser-252 or Ser-253 sites. The phosphorylation site is at Ser-271 in the case of the PIN4 protein. In the case of PIN7 protein, two phosphopeptides were detected in the digest, the Ser-431 and the Ser-277/Ser-278, respectively (Figure S3).

## 3. Discussion

### 3.1. The AtCRK5 Protein Kinase Controls the Gibberellin Level Influencing Seed Size and Embryogenesis

Ca^2+^ has a pivotal role as a secondary messenger in many abiotic and biotic stress responses in plants. Environmental cues trigger Ca^2+^ concentration alterations in plant cells that are perceived by calmodulin, several calcium-dependent protein kinases (CDPK superfamily members) and calcineurin B-like proteins [66,67,68,69,70,71,72,73]. The Calcium-Dependent Protein Kinase superfamily members (CDPKs) are among the main effectors of Ca^2+^ signal transduction in plants [71,72]. Members of the CDPK superfamily—including the Ca-dependent protein kinases (CDPKs), the CDPK-related protein kinases (CRKs), the Ca^2+^ and calmodulin-activated kinases (CCaMKs), and the sucrose non-fermenting1-related kinases3 (SnRK3s)—in addition to abiotic/biotic stress responses play important roles in many aspects of the regulation of plant growth and development [57,58,59,67,72,73,74]. In *Arabidopsis*, the CRK5 protein kinase is active in most *Arabidopsis* organs and it is certainly involved in the establishment of the proper auxin gradient necessary for gravitropic response of the root and for proper bending of the hypocotyl hook in Arabidopsis [57,59]. CRK5 is produced in the embryos at all embryo developmental stages and it is localised to the plasma membrane (Figure S4).

Here, we report a significant difference between the dry seed and embryo sizes of the wild type *Arabidopsis thaliana* (Col-0) and the At*crk5-1* mutant (Figure 1). These processes were investigated in more detail in the At*crk5-1* mutant to clarify the role of the AtCRK5 kinase in seed and embryo development. Microscopical investigation of the characteristic developmental stages of embryos developing within the siliques revealed that embryo development was strongly delayed in the At*crk5-1* mutant as compared to the wild type from the globular stage onward in the stage4–to–stage11 siliques (S4–to–S11). This embryo developmental period is related to the accumulation of bioactive gibberellins, GA_1_ and GA_3_. The accumulation of gibberellic acid in seeds is characterized with two-peaks that correspond to two specific stages of seed development. The first peak is associated with the stage of embryo differentiation when GA promotes cell growth and expansion (until heart embryo stage, S1–S6), and the second GA maximum takes place at the end of the maturation phase when gibberellins trigger proteolytic enzyme activity for the mobilization of endosperm resources for germination [3,4]. Bioactive gibberellins regulate late embryogenesis via modulating the DELLA protein level in the embryos.

GA deficiency and DELLA protein accumulation both lead to abnormal embryos characterized by a shortened embryo axis and unbending cotyledons [3]. This resembles to the phenotype of the late-stage At*crk5-1* mutant embryos: we also pointed out that the SAM-RAM distances are shorter in the embryos of the At*crk5-1* mutant as compared to the wild type ones and the cotyledons are unbended (Figure 4). Therefore, it was supposed that the observed delay in the embryogenesis of At*crk5-1* might be explained with the shortage or reduced level of bioactive GAs. In agreement, a decreased GA level could be measured in the medium sized (S4–S7) siliques of the At*crk5-1* mutant in comparison to the wild type. Moreover, the expressions of the key genes of GA metabolism were downregulated in the At*crk5-1* embryos during this developmental period (Figure 7F–H). We found that the treatment restored the SAM-RAM distance of the mutant embryos to the wild type level observed at S11–S12 stages when we employed 20 μM GA_3_ to the inflorescence of greenhouse-grown wild type *Arabidopsis* and At*crk5-1* mutant plants to investigate the effect of exogenous GAs on their embryogenesis (Figure 6A). Additionally, the delayed bending phenotype of the At*crk5-1* mutant embryos found in S10–S12 (Figure 4) was also restored to close to normal by the GA treatment (Figure 6C).

Our results indicate that the accumulation of GAs during the early phase of embryogenesis is missing or limited in the At*crk5-1* mutant embryos, which probably cause problems in embryo differentiation and delay the later embryo developmental stages (as depicted in Figure 2 and Figure 3).

### 3.2. AtCRK5 is a General Regulator of Auxin Distribution Potentially via the Phosphorylation of Several PINs

Auxin production, signaling, and the establishment of auxin gradients have a central role in embryo development and morphogenesis [4,9,10,11,12]. When considering the delay in the progression of embryogenesis in the At*crk5-1* mutant seeds, the distribution of the GFP-expressing auxin sensor DR5, and that of the GFP-tagged auxin transporters PIN1, PIN3, PIN4, PIN7, and AUX1 was also investigated during the whole process of embryogenesis in both phenotypes. We found a much weaker DR5::GFP signal in the At*crk5-1* embryos than in the control ones in all embryo developmental stages (Figure 8B), which indicated auxin shortage in the embryos. When the abundance of the auxin efflux transporter PIN proteins PIN1, PIN4, and PIN7 was checked in the At*crk5-1* embryos, we also observed strong decline in their level. We also found a low auxin influx protein (AUX1::YFP) level in the At*crk5-1* mutant in all of the studied embryo developmental stages. Auxin itself regulates the expression and stability of its transporters [75,76]. Therefore, a lower level of AUX1 might be the indirect consequence of PIN phosphorylation. The auxin influx carriers AUX1/LAX1/LAX2 have a crucial role in the formation of the embryo shoot and root poles, and they act redundantly to specify embryonic root and shoot pole identity and development [17,24], so any malfunction of these auxin transporters results in morphological changes in embryo shape. Actually, we found a reduction in the SAM-RAM distances of the At*crk5-1* embryos at the end of the embryogenesis (in S11–S12; Figure 4 and Figure 5), which is probably the consequence of the impaired cooperation of the auxin efflux and influx carrier’s PINs/AUXs in the absence of the AtCRK5 protein kinase. The auxin-dependent polarization of embryos necessitates balances of efflux and influx auxin transport mechanisms [17].

The phosphorylation status of these transporters determines the abundance and asymmetrical distribution of PIN proteins in the plasma membrane, which in turn affects the polar auxin transport [77]. Various kinases are known to control the distribution of auxin through the phosphorylation of the PIN efflux carriers or the ABC transporters [78]. Among others, the D6PROTEIN KINASE and PINOID/WAG AGCVIII kinases are types of kinases that are known to activate the PIN-mediated auxin efflux [65]. Previously, we have shown that the AtCRK5 kinase can phosphorylate in vitro the hydrophilic loops of various PIN proteins, like PIN2 and PIN3 [57,59]. Based on in vitro kinase assays and phospho-peptide analysis data, here we also report the possible phosphorylation sites of AtCRK5 in PIN1, PIN4, and PIN7. We assume that these transporters are also the targets of the AtCRK5 kinase. The AtCRK5-mediated phosphorylation of PIN proteins might increase their stability and maintain their optimal abundance at the various embryo stages. The role of AtCRK5 in the regulation of auxin transport controlling PIN protein abundance has been described during root gravitropic responses [57] and hypocotyl bending [59]. Our results suggest that AtCRK5 can have a general role maintaining the stability of several polar auxin transport (PAT) proteins, thus affecting auxin transport efficiency during various developmental processes, including embryogenesis, skotomorphogenesis, and gravitropism.

### 3.3. The AtCRK5 Protein Kinase is Involved in Hormonal Crosstalk Influencing Embryogenesis

Several data suggest that there is crosstalk between auxin transport and GA signaling [31,33,50,79]. It is already known that auxin promotes GA biosynthesis via the regulation of the expression of the two enzymes AtGA3ox and AtGA20ox being responsible for the rate limiting steps of GA biosynthesis [3,50]. This control mechanism is independent of the feedback regulation mediated by GAI and RGA DELLA proteins, and it involves Aux/IAA and ARF signaling elements [4,50]. It has also been pointed out that impaired auxin transport resulted in the stabilization of the DELLA repressor RGA (REPRESSOR OF ga1-3) of the GA pathway in *Arabidopsis* [80]. Therefore, the limited auxin transport in the At*crk5-1* embryos might be responsible for the observed reduction in their GA synthesis/level. Gibberellin shortage was shown to result in the formation of abnormal embryos at the S10–S11 silique stages with shortened embryo axis and unbending cotyledons [3]. The same characterizes the embryos of the At*crk5-1* mutant at this developmental stage. Abnormal embryo development of *Atcrk5-1* could be rescued by exogenous GA treatment (Figure 6), which indicated the primary role of GA shortage in the embryo phenotype of the mutant. Interestingly, we previously found an unclosed hypocotyl hook phenotype of At*crk5-1* seedlings during skotomorphogenesis, which might be explainable by the fact that the At*crk5-1* mutant embryos had already displayed reduced hypocotyl bending during their late embryogenesis ( Figure 1, Figure 2 and Figure 4 and [59]).

It was reported that the GA signaling pathway interacts with PIN-protein-dependent auxin transport in embryo cotyledon development and root gravitropism [33]. Auxin transport impairment was observed in the GA biosynthesis and signaling mutant *ga1,* which was explained by a decrease of PIN auxin efflux carrier abundance [33]. GA deficiency resulted in targeting PIN2 for vacuolar degradation, leading to reduction of auxin transport. The PIN protein levels could be restored in *ga1* to the wild type level by adding exogenous GA. Therefore, the auxin transport impairment in the At*crk5-1* embryos might be related to the impaired GA biosynthesis, resulting in GA deficiency, similarly as it was found in [33].

**Figure 14:**
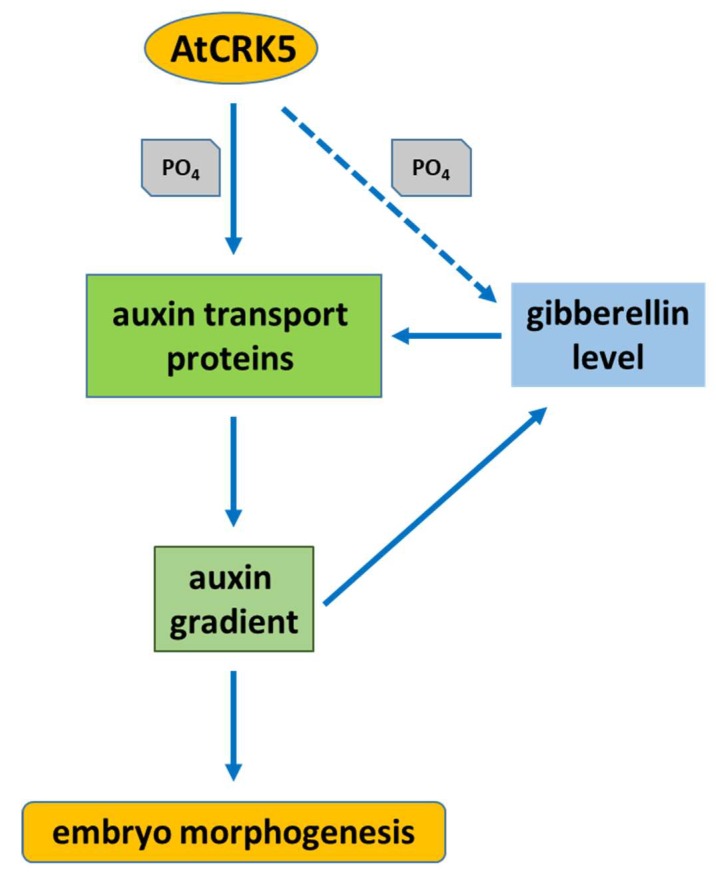
Hypothetical model about the involvement of the AtCRK5 protein kinase in the coordination of embryo morphogenesis in *Arabidopsis thaliana*. The auxin transport proteins having central role in the establishment of auxin gradients controlling embryo morphogenesis are potential targets of AtCRK5. The phosphorylation of these transporters by AtCRK5 increases their stability. In the absence of AtCRK5 their level is decreased without affecting their distribution pattern. The auxin gradients therefore are properly established in the At*crk5-1* mutant but at lower auxin maxima in the corresponding regions. The lower auxin concentration in these regions limits gibberellin synthesis, via the decreased expression of key genes of gibberellin metabolisms. The lower GA level results in lower expression of the genes coding for auxin transport proteins strengthening the phenotype. An auxin-transport-independent effect of the AtCRK5 kinase on gibberellin metabolism cannot be excluded. In summary, in the absence of AtCRK5, embryogenesis progresses at correctly organised but lower auxin and gibberellin levels delaying but not preventing the processes of morphogenesis.

Our results suggest that, AtCRK5 can also govern the embryo development in *Arabidopsis* through the fine tuning of auxin-GA levels via the stability/abundance of the polar auxin transport (PAT) proteins in addition to its regulatory role in root gravitropic responses and hypocotyl bending [57,59], as indicated in Figure 14.

## 4. Materials and Methods

### 4.1. Plant Material and Growth Conditions

All of the plants used in this study are in *Arabidopsis thaliana* (L.) Columbia-0 ecotype (Col-0) background. The At*crk5-1* mutant has been described previously [57,58]. The auxin sensor DR5::GFP [61], AtCRK5-GFP [57], PIN1:PIN1-GFP [81], PIN3:PIN3-GFP [82], PIN4:PIN4-GFP [83], PIN7:PIN7-GFP [83], and AUX1::YFP [84] constructs were also described in [57]. All the PAT-GFP proteins and CRK5-GFP were driven by their own genomic promoters. We introduced the DR5::GFP and PIN1:PIN1-GFP, PIN4:PIN4-GFP, and PIN7:PIN7-GFP into wild type (Col-0) and mutant (At*crk5-1*) backgrounds via deep floral transformation [57,85]. Sexual crossings introduced PIN3:PIN3-GFP and AUX1::YFP into wild type and mutant backgrounds [57]. Generally, wild type and mutant seeds were sterilized and kept at 4 °C for two days, as described in [57,59].

When it was necessary, the imbibed seeds were transferred onto plates containing half strength Murashige and Skoog medium (1/2 MS) with 0.5% sugar (Molar Chemicals Kft, Hungary), 0.8% phytoagar (Duchefa Biochemie, Haarlem, The Netherlands), pH: 5.7. After seed transfer (AST), the plates were kept in white light for 5 h to stimulate and synchronize seed germination The plates were put into short day condition (SD, 8h light/16h dark cycle, 22 °C, 100 μmol photons m^−2^ s^−1^ light intensity). For GA rescue experiments, the developing siliques on the inflorescences of one-month-old greenhouse plants were sprayed once in every day with 20 μM GA_3_ (Phylaxia, Budapest, Hungary) until silique number 14 (S14) was obtained.

### 4.2. Seed/Embryo Size/Axis Determination, Embryo Isolation and GA Rescue Experiments

The wild type Col-0 and At*crk5-1* seeds were sterilized in a standard manner and stored in vials for two days at 4 °C for seed/embryo size determination. 100 μL seeds from each vial were taken out onto a slide and covered with cover slips. Seed sizes (perimeter) in the intact seeds were quantified in wild type (Col-0) and mutant (At*crk5-1)* lines after two days of imbibition at 4 °C by photographing the seeds with a stereo microscope Nikon SMZ800 (Mitsubishi Corp., Minato, Tokyo, Japan) attached to a CCD camera (Nikon Cool pix 995, Japan). The photographs were then evaluated with the ImageJ software (NIH, Bethesda, MD, USA; (https://imagej.net/Fiji/Downloads)). The average values were taken from minimum 100 independent seeds from the wild type and mutant genotypes, respectively.

Embryo axis length, as measured from shoot apical meristem (SAM) until root apical meristem (RAM), was calculated in bent-cotyledon-stage embryos that arose from the previously described experiment. ImageJ measured at least 100 embryo axes. The differences in hook angles of wild type Col-0 and mutant At*crk5-1* embryos after two days of imbibition at 4 °C were also measured. The bent-cotyledon-stage embryos were carefully pressed out from the seed coat by forceps under a stereo microscope Nikon SMZ800 (Mitsubishi Corp., Minato, Tokyo, Japan) and they were photographed by a CCD camera (Nikon Cool pix 995, Japan). Images were evaluated by the ImageJ software (NIH, Bethesda, MD, USA). The apical hook angles were defined according to the inset in Figure 1C. At least 100 wild-type and mutant seedlings were finally monitored in three biological repeats. Student’s t-test was used for statistical analysis for all quantitative measurements. All of the experiments was repeated three times.

### 4.3. Total GA Measurement by Competitive GAs Elisa Assay

The septum bearing the seeds without valves that originated from medium sized siliques (S5–S7 from the top of inflorescence) were collected from six weeks old greenhouse grown wild type (Col-0) and mutant (At*crk5-1*) plants in the case of total GA content measurement from siliques arised seeds. The seeds were dissected from the siliques under stereo microscope Nikon SMZ800 (Mitsubishi Corp., Minato, Tokyo, Japan) and they were collected into Eppendorf tubes on dry ice. We used Retsch (Haan, Germany) tissuelyzer for grind the samples. The grounded samples were incubated overnight at 4 °C in 10mL of 80% methanol solution. After centrifugation with 12,000 rpm, 10 min. at 4 °C, the supernatants were transferred to new tubes, and they were completely lyophilized. We added 200 µl PBS to each sample and loaded 50 µl/well. Three technical repetitions were used. For ELISA measurement, we followed the protocol of the kit (ELISA Kit for Gibberellic Acid (GA), CEA759Ge, Cloud Clone Corp. Wuhan, China). Three biological repeats were performed for total GA content evaluation.

### 4.4. Embryo Morphology Monitored by Cell-R Microscopy

Twelve-twelve siliques (S1–S11/12 where S1 represents the youngest and S12 the oldest siliques after pollination; silique position number) with various developmental stages of embryos were collected from wild type Col-0 and At*crk5-1* inflorescence of greenhouse plants. The seeds were dissected from siliques and, after preclearing—depending on the age of the siliques—they were incubated in Hoyer’s solution for 4 h (for small siliques) or overnight (for medium and large siliques) [86]. Subsequently, seed developmental stages were determined by differential interference contrast microscope (Cell-R, Olympos Corporation, Tokyo, Japan).

For GA rescue experiments, the developing siliques on inflorescences of one-month greenhouse plants were sprayed once in every day with 20 μM GA_3_ (Phylaxia, Budapest, Hungary) until silique number 14 (S14) was obtained. Afterwards, siliques were harvested and handled as described above.

### 4.5. Monitoring of Abundance of GFP/YFP Signals in Embryos by LSM Microscopy

The seeds of the GFP/YFP tagged lines of wild type Col-0 and At*crk5-1* plants of various developmental stages were collected into 10% (*v*/*v*) glycerol from several silique stages from developing inflorescences of plants. Embryos were cautiously pressed out from the seeds by using a mild pressure of the glass cover. Afterwards, the abundance/distribution of GFP/YFP signals in the case of various stages of embryogenesis for the auxin sensor DR5::GFP, the auxin transporters (PIN1-GFP, PIN3-GFP, PIN4-GFP, PIN7-GFP, and AUX1::YFP), and the AtCRK5-GFP was analysed while using Olympus FV1000 confocal laser scanning microscopy (Tokyo, Japan). The fluorescence signals were monitored according to [57,58,59]. Generally, 5-5 embryos were investigated for both categories (wild type and mutant) from three independent experiments. The images were prepared while using the Adobe Photoshop and Illustrator software (Adobe Systems Incorporated, San Jose, CA, USA).

### 4.6. RNA Isolation and Real Time Quantitative PCR (qRT-PCR) for Embryo Gene Expression

The isolation of RNA was performed with a slightly modified CTAB-LiCl extraction method of [87]: 100 mg material was collected from 45-days-old green-house-grown plants from small (S1–S4), medium (S5–S7), and large (S8–S10) siliques of Col-0 and At*crk5-1,* respectively. cDNA synthesis of 1 μg of total RNA was carried out in 20 μL using RevertAid M-MuLV Reverse Transcriptase (Thermo Fischer Scientific, Waltham, MA, USA). Quantitative real time PCR (qRT-PCR) was carried out applying the SYBR Green master mix (Applied Biosystems, Thermo Fischer Scientific, Vilnius, Lithuania) with the ABI 7900 Fast Real Time System (Applied Biosystems, Thermo Fischer Scientific, Vilnius, Lithuania) while following the next protocol: 45 cycles at 95 °C for 15 s, followed by 60 °C for 1 min. The 2^−ΔΔCt^ method calculated the normalized relative transcript levels [88]. Reactions were made in triplicates and minimum two independent biological repetitions were performed. *Ubiquitin-1* was used as an endogenous control. Table S1 lists the set for qRT PCR primers used in this study.

### 4.7. PIN4 and PIN7 Hydrophilic Loop Region Cloning

We amplified the corresponding cDNA sequences from *Arabidopsis thaliana* cDNAs applying the high fidelity Phusion polymerase (Thermo Fischer Scientific, Vilnius, Lithuania) while following the manufacturer’s instructions (Figure S5). Table S1 lists the specific primers used. After amplification, the BamHI-EcoRI enzyme-digested cDNA fragment was ligated into the pBluescript II SK plasmid. Sequencing justified the error free cloned fragments. The PIN1HL loop fragment was moved into the pGEX4T1 (Amersham, Little Chalfont, Great Britain) protein expression vector, while the PIN4HL and PIN7HL loop fragments were moved into the pMALp2 (New England Biolabs, Ipswich, MA, USA) protein expression vector, to get in frame fusion with the N-terminal GST (in the case of PIN1HL) or MBP (in the case of PIN4 and PIN7) tag.

### 4.8. Purification of Tagged Proteins

All of the protein expression constructs were transformed into BL21DE3Rosetta (Novagen part of Merck KGaA, Darmstadt, Germany) competent cells, streaked into LB media supplemented with 100 mg/L carbenicillin, 34 mg/L chloramphenicol, and 1% of glucose, and the plates were then incubated o/n at 37 °C. For expression and purification of proteins, we followed the manufacturer’s instructions, e.g., in the case of pGEX4T1-PIN1HL loop, the GST phusion protein protocol from Amersham (Amersham, Little Chalfont, Great Britain), while in the case of the pMALp2-PIN4HL loop and pMALp2-PIN4HL loop, we used the MBP phusion protein protocol from New England Biolabs (Ipswich, Massachusetts, USA). After the elution of the recombinant proteins, we checked the fractions by 10% SDS-polyacrylamide gel electrophoresis (SDS-PAGE). The fractions containing most of the recombinant proteins were identified, pooled, and dialyzed (10 mM Tris-HCl (pH 7.5), 50 mM NaCl, 10% glycerin and 5 mM 2-mercaptoethanol) at 4 °C, and then stored at −80 °C for later use.

### 4.9. In vitro Kinase Assays

The in vitro kinase assays were performed as described in [57]. We used 1 µg His_6_-CRK5 kinase in 20 µL kinase buffer (20 mM Tris-HCl [pH 8.0], 5mM MgCl_2_, 1mM DTT, and 5 µCi [γ-32P] ATP) containing 2 µg Myelin BasicProtein (MPB), Sigma-Aldrich (St. Louis, Missouri, USA) as a control kinase substrate, or the GST-PIN1HL loop as substrate. The reaction was performed at room temperature for 30–45 min. and stopped by adding 1× Laemmli SDS sample buffer, boiled, and then size-separated by 10% SDS-PAGE. After staining with Coomassie dye, the gel was then subjected to autoradiography using X-ray film.

### 4.10. Mass Spectrometry

For mass spectrometry assay, we used about 1 µg of purified His_6_-CRK5 kinase and 1-1 µg GST-PIN1HL, MBP-PIN4HL, or MBP-PIN7HL as substrates. We performed non-radioactive in vitro kinase assay in 20 μL kinase buffer (20 mM Tris-HCl [pH 8.0], 5mM MgCl_2_, 1mM DTT) supplemented with 1mM ATP (Thermo Scientific, Waltham, Massachusetts, USA). The reaction was incubated at room temperature for 45–60 min. and then stopped by adding 1X Laemmli SDS sample buffer. The mixture was boiled and size-separation by SDS-PAGE. After staining with Coomassie dye, the stained GST-PIN1HL, MBP-PIN4HL, and MBP-PIN7HL protein bands were excised and analysed by mass spectrometry in the Laboratory of Proteomics Research of the BRC, Szeged, Hungary (http://www.brc.hu/core_proteomics_research.php). Small aliquots of the tryptic digests were retained and the rest were subjected to phosphopeptide enrichment by immobilized metal affinity chromatography on Fe-NTA magnetic agarose beads (that were prepared from Ni(II)-NTA Magnetic Agarose Beads, Qiagen, Hilden, Germany). LC-MS/MS analysis of the digests with and without phosphopeptide enrichment was performed on a Thermo LTQ Orbitrap Fusion Lumos mass spectrometer (Thermo Scientific, Waltham, Massachusetts, USA) in data dependent mode applying HCD fragmentation, followed by database search against the sequences of the fusion proteins appended to *E. coli* entries in the Uniprot 2015.4.16 (GST-PIN1HL) and 2019.6.12 (MBP-PIN4HL and MBP-PIN7HL) database. The results of the database searches were manually validated.

### 4.11. Bioinformatic Analysis and Tools

Primers were prepared while using the Primer3Plus software (http://www.bioinformatics.nl/cgi-bin/primer3plus/primer3plus.cgi). For DNA manipulation, the VectorNTI (Thermo Fisher Scientific, Waltham, MA, USA) and Lasergene (DNAStar Inc., Madison, WI, USA) program suits were used. The ABI SDS software (Thermo Fischer Scientific, Waltham, Massachusetts, USA) was used to analyze the specificity of the amplifications of the genes for expression by qRT-PCR.

### 4.12. Accession Numbers

Sequence data used in this study can be found in the Arabidopsis Information Resource (TAIR) and GenBank (NCBI) databases under the following accession numbers: *AtCRK5 (At3g50530*), *PIN1* (*At1g73590*), *PIN3* (*At3g70940*), *PIN4* (*At2g01420*), *PIN7* (*At1g23080*), *AUX1* (*At2g38120*), *RGL1* (*At1g66350*), *RGL2* (*At3g0345*), *RGL3* (*At5g17490*), *GAI* (*At1g14920*), *RGA* (*At2g01570*), *GA20ox2* (*At5g51810*), *GA20ox3* (*At5g07200*), *GA2ox4* (*At1g60980*), *LEC1* (*At1g21970*), *LEC2* (*At1g28300*), *FUS3* (*At3g26790*), *ABI3* (*At3g24650*), and *Ubiquitin1* (*UBQ-1*, *At3g52590*).

### 4.13. Statistical Analysis

The experiments were carried out with two or three independent biological repetitions, as indicated. Sample numbers are given for each experiment in the text/figure legends. The data are presented as the mean ± standard error (SE) calculated from the combined data of biological repetitions. Differences of the mutant from the wild type control were determined by Student’s t-test and the significant differences were represented, as follows: * *p* ≤ 0.05; ** *p* ≤ 0.01; *** *p* ≤ 0.001.

## Supplementary Materials

Supplementary materials can be found at https://www.mdpi.com/1422-0067/20/24/6120/s1.

## References

[1] Mayer U., Buttner G., Jurgens G. (1993). Apical-basal pattern formation in the Arabidopsis embryo: Studies on the role of the gnom gene. Development.

[2] Ten Hove C.A., Lu K.-J., Weijers D. (2015). Building a plant: Cell fate specification in the early *Arabidopsis* embryo. Development.

[3] Hu Y., Zhou L., Huang M., He X., Yang Y., Liu X., Li Y., Hou X. (2018). Gibberellins play an essential role in late embryogenesis of Arabidopsis. Nat. Plants.

[4] Locascio A., Roig-Villanova I., Bernardi J., Varotto S. (2014). Current perspectives on the hormonal control of seed development in Arabidopsis and maize: A focus on auxin. Front. Plant Sci..

[5] Teale W.D., Paponov I.A., Palme K. (2006). Auxin in action: Signalling, transport and the control of plant growth and development. Nat. Rev. Mol. Cell Biol..

[6] Vanneste S., Friml J. (2009). Auxin: A trigger for change in plant development. Cell.

[7] Vanstraelen M., Benkova E. (2012). Hormonal interactions in the regulation of plant development. Annu. Rev. Cell Dev. Biol..

[8] Zádnikova P., Smet D., Zhu Q., Van der Straeten D., Benková E. (2015). Strategies of seedlings to overcome their sessile nature: Auxin in mobility control. Front. Plant Sci..

[9] Hamann T., Benkova E., Baurle I., Kientz M., Jurgens G. (2002). The Arabidopsis BODENLOS gene encodes an auxin response protein inhibiting MONOPTEROS-mediated embryo patterning. Genes Dev..

[10] Friml J., Vieten A., Sauer M., Weijers D., Schwarz H., Hamann T., Offringa R., Jürgens G. (2003). Efflux-dependent auxin gradients establish the apical-basal axis of Arabidopsis. Nature.

[11] Jenik P.D., Barton M.K. (2005). Surge and destroy: The role of auxin in plant embryogenesis. Development.

[12] Wabnik K., Robert H.S., Smith R.S., Friml J. (2013). Modeling framework for the establishment of the apical-basal embryonic axis in plants. Curr. Biol..

[13] Bennett M.J., Marchant A., Green H.G., May S.T., Ward S.P., Millner P.A., Walker A.R., Schulz B.F., Feldmann K.A. (1996). Arabidopsis AUX1 gene: A permease-like regulator of root gravitropism. Science.

[14] Petrasek J., Mravec J., Bouchard R., Blakeslee J.J., Abas M., Seifertova D., Wiśniewska J., Tadele Z., Kubeš M., Čovanová M. (2006). PIN proteins perform a rate-limiting function in cellular auxin efflux. Science.

[15] Krecek P., Skupa P., Libus J., Naramoto S., Tejos R., Friml J. (2009). The PIN-FORMED (PIN) protein family of auxin transporters. Genome Biol..

[16] Barbez E., Kubeš M., Rolčík J., Béziat C., Pěnčík A., Wang B., Rosquete M.R., Zhu J., Dobrev P.I., Lee Y. (2012). A novel putative auxin carrier family regulates intracellular auxin homeostasis in plants. Nature.

[17] Robert H.S., Grunewald W., Sauer M., Cannoot B., Soriano M., Swarup R., Weijers D., Bennett M., Boutilier K., Friml J. (2015). Plant embryogenesis requires AUX/LAX-mediated auxin influx. Development.

[18] Adamowski M., Friml J. (2015). PIN-dependent auxin transport: Action, regulation and evolution. Plant Cell.

[19] Liu Y., Dong Q., Kita D., Huang J.B., Liu G., Wu X., Zhu X., Cheung A.Y., Wu H.M., Tao L.Z. (2017). RopGEF1 Plays a Critical Role in Polar Auxin Transport in Early Development. Plant Physiol..

[20] Zhou J.J., Luo J. (2018). The PIN-FORMED auxin effux carriers in plants. Int. J. Mol. Sci..

[21] Wisniewska J., Xu J., Seifertova D., Brewer P.B., Ruzicka K., Blilou I., Rouquie D., Benkova E., Scheres B., Friml J. (2006). Polar PIN localization directs auxin flow in plants. Science.

[22] Möller B., Weijers D. (2009). Auxin Control of Embryo Patterning. Cold Spring Harb. Perspect. Biol..

[23] Robert H.S., Chulmin Park C., Gutierrez C.L., Wójcikowska B., Pěnčík A., Novák O., Chen J., Grunewald W., Dresselhaus T., Friml J. (2018). Maternal auxin supply contributes to early embryo patterning in *Arabidopsis*. Nat. Plants.

[24] Ugartechea-Chirino Y., Swarup Y.R., Swarup K., Peret B., Whitworth M., Bennett M., Bougourd S. (2010). The AUX1 LAX family of auxin influx carriers is required for the establishment of embryonic root cell organization in *Arabidopsis thaliana*. Ann. Bot..

[25] Swain S.M., Reid J.B., Kamiya Y. (1997). Gibberellins are required for embryo growth and seed development in pea. Plant J..

[26] Hays D.B., Yeung E.C., Pharis R.P. (2002). The role of gibberellins in embryo axis development. J. Exp. Bot..

[27] Singh D.P., Jermakow A.M., Swain S.M. (2002). Gibberellins are required for seed development and pollen tube growth in *Arabidopsis*. Plant Cell.

[28] Sun T.P. (2008). Gibberellin metabolism, perception and signaling pathways in *Arabidopsis*. Arab. Book.

[29] Yamaguchi S. (2008). Gibberellin metabolism and its regulation. Annu. Rev. Plant Biol..

[30] Schwecheimer C. (2012). Gibberellin signaling in plants—The extended version. Front. Plant Sci..

[31] Abbas M., Alabadi D., Blazquez M.A. (2013). Differential growth at the apical hook: All roads lead to auxin. Front. Plant Sci..

[32] Salanenka Y., Verstraeten I., Löfke C., Tabata K., Naramoto S., Glanc M., Friml J. (2018). Gibberellin DELLA signaling targets the retromer complex to redirect protein trafficking to the plasma membrane. Proc. Natl. Acad. Sci. USA.

[33] Willige B.C., Isono E., Richter R., Zourelidou M., Schwechheimer C. (2011). Gibberellin Regulates PIN-FORMED Abundance and Is Required for Auxin Transport–Dependent Growth and Development in *Arabidopsis thaliana*. Plant Cell.

[34] Sun T.P., Kamiya Y. (1994). The Arabidopsis GAl Locus Encodes the Cyclase ent-Kaurene Synthetase A of Gibberellin Biosynthesis. Plant Cell.

[35] Willige C.V., Ghosh S., Nill C., Zourelidou M., Dohmann E.M.N., Maier A., Schwechheimer C. (2007). The DELLA Domain of GA INSENSITIVE Mediates the Interaction with the GA INSENSITIVE DWARF1A Gibberellin Receptor of Arabidopsis. Plant Cell.

[36] Sreenivasulu N., Wobus U. (2013). Seed-development programs: A systems biology-based comparison between dicots and monocots. Annu. Rev. Plant Biol..

[37] Ye N., Zhang J. (2012). Antagonism between abscisic acid and gibberellins is partially mediated by ascorbic acid during seed germination in rice. Plant Signal. Behav..

[38] Liu X., Hou X. (2018). Antagonistic Regulation of ABA and GA in Metabolism and Signaling Pathways. Front. Plant Sci..

[39] Shu K., Zhou W., Chen F., Luo X., Yang W. (2018). Abscisic Acid and Gibberellins Antagonistically Mediate Plant Development and Abiotic Stress Responses. Front. Plant Sci..

[40] Koornneef M., Jorna M.L., Brinkhorst-Van Der Swan D.L., Karssen C.M. (1982). The isolation of abscisic acid (ABA) deficient mutants by selection of induced revertants in non-germinating gibberellin sensitive lines of *Arabidopsis thaliana* (L.) heynh. Theor. Appl. Genet..

[41] Roscoe T.T., Guilleminot J., Bessoule J.-J., Berger F., Devic M. (2015). Complementation of Seed Maturation Phenotype by Ectopic Expression of ABSCISIC ACID INSENSITIVE3, FUSCA3 and LEAFY COTYLEDON2 in Arabidopsis. Plant Cell Physiol..

[42] West M.A.L., Yee K.M., Danao J., Zimmermann J.L., Fischer R.L., Goldberg R.B., Harada J.J. (1994). LEAFY COTYLEDON1 is an essential regulator of late embryogenesis and cotyledon identity in *Arabidopsis*. Plant Cell.

[43] Luerssen H., Kirik V., Herrmann P., Misera S. (1998). FUSCA3 encodes a protein with a conserved VP1/ABI3-like B3 domain which is of functional importance for the regulation of seed maturation in *Arabidopsis thaliana*. Plant J..

[44] Stone S.L., Kwong L.W., Yee K.M., Pelletier J., Lepiniec L., Fischer R.L., Goldberg R.B., Harada J.J. (2001). LEAFY COTYLEDON2 encodes a B3 domain transcription factor that induces embryo development. Proc. Natl. Acad. Sci. USA.

[45] Lee H., Fischer R.L., Goldberg R.B., Harada J.J. (2003). Arabidopsis LEAFYCOTYLEDON1 represents a functionally specialized subunit of the CCAAT binding transcription factor. Proc. Natl. Acad. Sci. USA.

[46] Braybrook S.A., Harada J.J. (2008). LECs go crazy in embryo development. Trends Plant Sci..

[47] Yamamoto A., Yoshii M., Murase S., Fujita M., Kurata N., Hobo T., Kagaya Y., Takeda S., Hattori T. (2014). Cell-by-Cell Developmental Transition from Embryo to Post-Germination Phase Revealed by Heterochronic Gene Expression and ER-Body Formation in *Arabidopsis leafy* cotyledon Mutants. Plant Cell Physiol..

[48] Cheng Y., Dai X., Zhao Y. (2007). Auxin synthesized by the YUCCA flavin monooxygenases is essential for embryogenesis and leaf formation in *Arabidopsis*. Plant Cell.

[49] Liu X., Zhang H., Zhao Y., Feng Z., Li Q., Yang H.Q., Luan S., Li J., He Z.H. (2013). Auxin controls seed dormancy through stimulation of abscisic acid signaling by inducing ARF-mediated ABI3 activation in Arabidopsis. Proc. Natl. Acad. Sci. USA.

[50] Frigerio M., Alabadı D., APerez-Gomez J., Garcıa-Carcel L., Phillips A.F., Hedden P., Blazquez M.A. (2006). Transcriptional regulation of gibberellin metabolism genes by auxin signaling in Arabidopsis. Plant Physiol..

[51] Rieu I., Ruiz-Rivero O., Fernandez-Garcia N., Griffiths J., Powers S.J., Gong F., Linhartova T., Eriksson S., Nilsson O., Thomas S.G. (2008). The gibberellin biosynthetic genes AtGA20ox1 and AtGA20ox2 act, partially redundantly, to promote growth and development throughout the Arabidopsis life cycle. Plant J..

[52] Dorcey E., Urbez C., Blazquez M.A., Carbonell J., Perez-Amador M.A. (2009). Fertilization-dependent auxin response in ovules triggers fruit development through the modulation of gibberellin metabolism in Arabidopsis. Plant J..

[53] Peng J., Carol P., Richards D.E., King K.E., Cowling R.J., Murphy G.P., Harberd N.P. (1997). The *Arabidopsis* GAI gene defines a signaling pathway that negatively regulates gibberellin responses. Genes Dev..

[54] Silverstone A.L., Ciampaglio C.N., Sun T. (1998). The *Arabidopsis* RGA gene encodes a transcriptional regulator repressing the gibberellin signal transduction pathway. Plant Cell.

[55] Lee S., Cheng H., King K.E., Wang W., He Y., Hussain A., Lo L., Harberd N.P., Peng J. (2002). Gibberellin regulates *Arabidopsis* seed germination via RGL2, a GAI/RGA-like gene whose expression is up-regulated following imbibition. Genes Dev..

[56] Daviere J.M., Achard P. (2016). A pivotal role of DELLAs in regulating multiple hormone signals. Mol. Plant.

[57] Rigó G., Ayaydin F., Tietz O., Zsigmond L., Kovács H., Páy A., Salchert K., Darula Z., Medzihradszky K.F., Szabados L. (2013). Inactivation of plasma membrane-localized CDPK-RELATED KINASE5 decelerates PIN2 exocytosis and root gravitropic response in Arabidopsis. Plant Cell.

[58] Baba A.I., Rigó G., Ayaydin F., Rehman A.U., Andrási N., Zsigmond L., Valkai I., Urbancsok J., Vass I., Pasternak T. (2018). Functional Analysis of the Arabidopsis thaliana CDPK-Related Kinase Family: AtCRK1 Regulates Responses to Continuous Light. Int. J. Mol. Sci..

[59] Baba A.I., Andrási N., Valkai I., Gorcsa T., Koczka L., Darula Z., Medzihradszky K.F., Szabados L., Fehér A., Rigó G. (2019). AtCRK5 Protein Kinase Exhibits a Regulatory Role in Hypocotyl Hook Development during Skotomorphogenesis. Int. J. Mol. Sci..

[60] Robert S.H. (2019). Molecular communication for coordinated seed and fruit dvelopment: What can we learn from auxin and sugars?. Int. J. Mol. Sci..

[61] Ottenschläger I., Wolff P., Wolverton C., Bhalerao R.P., Sandberg G., Ishikawa H., Evans M., Palme K. (2003). Gravity-regulated differential auxin transport from columella to lateral root cap cells. Proc. Natl. Acad. Sci. USA.

[62] Jenik P.D., Gillmor C.S., Lukowitz W. (2007). Embryonic patterning in *Arabidopsis thaliana*. Annu. Rev. Cell Dev. Biol..

[63] Offringa R., Huang F. (2013). Phosphorylation-dependent trafficking of plasma membrane proteins in animal and plant cells. J. Integr. Plant Biol..

[64] Luschnig C., Vert G. (2014). The dynamics of plant plasma membrane proteins: PINs and beyond. Development.

[65] Barbosa I.C.R., Hammes U.Z., Schwechheimer C. (2018). Activation and polarity control of PIN-FORMED auxin transporters by phosphorylation. Trends Plant Sci..

[66] Hrabak E.M., Chan C.W.M., Gribskov M., Harper J.F., Choi J.H., Halford N., Kudla J., Luan S., Nimmo H.G., Sussman M.R. (2003). The *Arabidopsis* CDPK-SnRK Superfamily of Protein Kinases. Plant Physiol..

[67] Harper J.F., Breton G., Harmon A. (2004). Decoding Ca^2^^+^ signals through plant protein kinases. Annu. Rev. Plant Biol..

[68] Zhang L., Lu Y.-T. (2003). Calmodulin-binding protein kinases in plants. Trends Plant Sci..

[69] Zhang L., Du L., Poovaiah B.W. (2014). Calcium signaling and biotic defense responses in plants. Plant Signal Behav..

[70] Zeng H., Xu L., Singh A., Wang H., Du L., Poovaiah B.W. (2015). Involvement of calmodulin and calmodulin-like proteins in plant responses to abiotic stresses. Front Plant Sci..

[71] Wang J.P., Xu Y.P., Munyampundu J.P., Liu T.Y., Cai X.Z. (2016). Calcium dependent protein kinase (CDPK) and CDPK related kinase (CRK) gene families in tomato: Genome wide identification and functional analyses in disease resistance. Mol. Genet. Genom..

[72] Simeunovic A., Mair A., Wurzinger B., Teige M. (2016). Know where your clients are: Subcellular localization and targets of calcium-dependent protein kinases. J. Exp. Bot..

[73] Delormel T.Y., Boudsocq M. (2019). Properties and functions of calcium dependent protein kinases and their relatives in *Arabidopsis thaliana*. New Phytol..

[74] Baba A.I., Rigó G., Andrási N., Tietz O., Palme K., Szabados L., Cséplő Á., Palócz-Andresen M., Szalay D., Gosztom A., Sípos L., Taligás T. (2019). Striving Towards Abiotic Stresses: Role of the Plant CDPK Superfamily Members. International Climate Protection.

[75] Leyser O. (2006). Dynamic integration of auxin transport and signalling. Curr. Biol..

[76] Paponov I.A., Paponov M., Teale W., Menges M., Chakrabortee S., Murray J.A.H., Palme K. (2008). Comprehensive transcriptome analysis of auxin responses in Arabidopsis. Mol. Plant.

[77] Ganguly A., Sasayama D., Cho H.T. (2012). Regulation of the polarity of protein trafficking by phosphorylation. Mol. Cells..

[78] Barbosa I.C.R., Schwechheimer C. (2014). Dynamic control of auxin transport-dependent growth by AGCVIII protein kinases. Curr. Opin. Plant Biol..

[79] Mazzella M.A., Casal J.J., Muschietti J.P., Fox A.R. (2014). Hormonal networks involved in apical hook development in darkness and their response to light. Front. Plant Sci..

[80] Fu X., Harberd N.P. (2003). Auxin promotes *Arabidopsis* root growth by modulating gibberellin response. Nature.

[81] Benková E., Michniewicz M., Sauer M., Teichmann T., Seifertová D., Jürgens G., Friml J. (2003). Local efflux-dependent auxin gradients as a common module for plant organ formation. Cell.

[82] Zadnikova P., Petrasek J., Marhavy P., Raz V., Vandenbussche F., Ding Z., Schwarzerová K., Morita M.T., Tasaka M., Hejátko J. (2010). Role of PIN-mediated auxin efflux in apical hook development of *Arabidopsis thaliana*. Development.

[83] Blilou I., Xu J., Wildwater M., Willemsen V., Paponov I., Friml J., Heidstra R., Aida M., Palme K., Scheres B. (2005). The PIN auxin efflux facilitator network controls growth and patterning in Arabidopsis roots. Nature.

[84] Swarup R., Friml J., Marchant A., Ljung K., Sandberg G., Palme K., Bennett M. (2001). Localization of the auxin permease AUX 1suggests two functionally distinct hormone transport pathways operate in the Arabidopsis root apex. Genes Dev..

[85] Bechtold N., Ellis J., Pelletier G. (1993). In planta Agrobacterium mediated gene transfer by infiltration of adult *Arabidopsis thaliana* plants. C. R. Acad. Sci. Paris Life Sci..

[86] Stangeland B., Salehian Z. (2002). An Improved Clearing Method for GUS Assay in *Arabidopsis* Endosperm and Seeds. Plant Mol. Biol. Rep..

[87] Jaakola L., Pirttilä A., Halonen M., Hohtola A. (2001). Isolation of high quality RNA from bilberry (*Vaccinium myrtillus L*.) fruit. Mol. Biotechnol.

[88] Czechowski T., Stitt M., Altmann T., Udvardi M.K., Scheible W.R. (2005). Genome-Wide Identification and Testing of Superior Reference Genes for Transcript Normalization in Arabidopsis. Plant Physiol..

