# Supplementary figures and images for: CRK5 Protein Kinase Contributes to the Progression of Embryogenesis of Arabidopsis thaliana
Source: Int J Mol Sci. 2019 Dec 4;20(24):6120. doi: 10.3390/ijms20246120 (PMC6941128; doi:10.3390/ijms20246120)

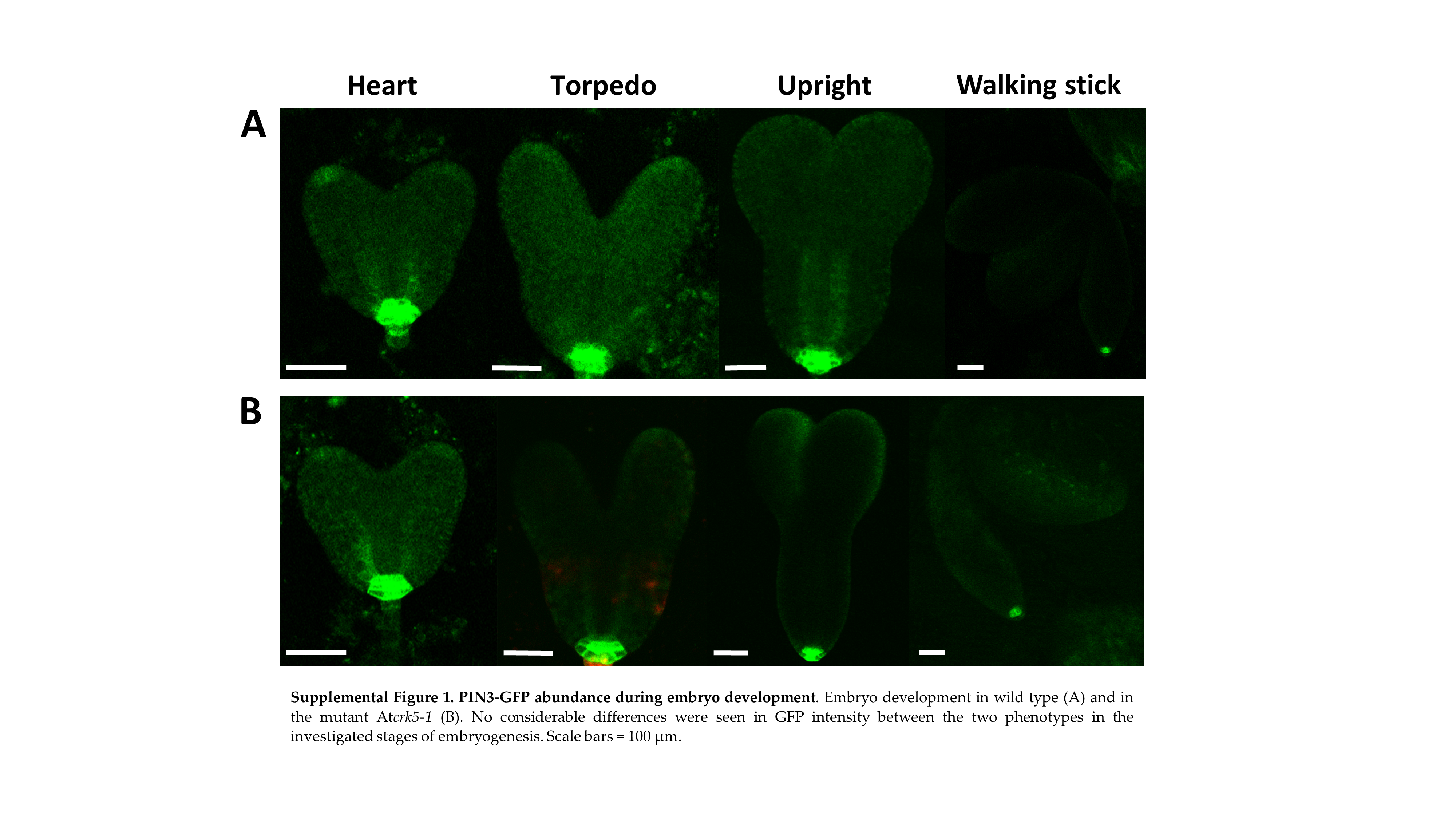

Supplement: Supplementary file 1 [file ijms-20-06120-s001.zip › Supplementary/Supplemental Figure 1.tif]

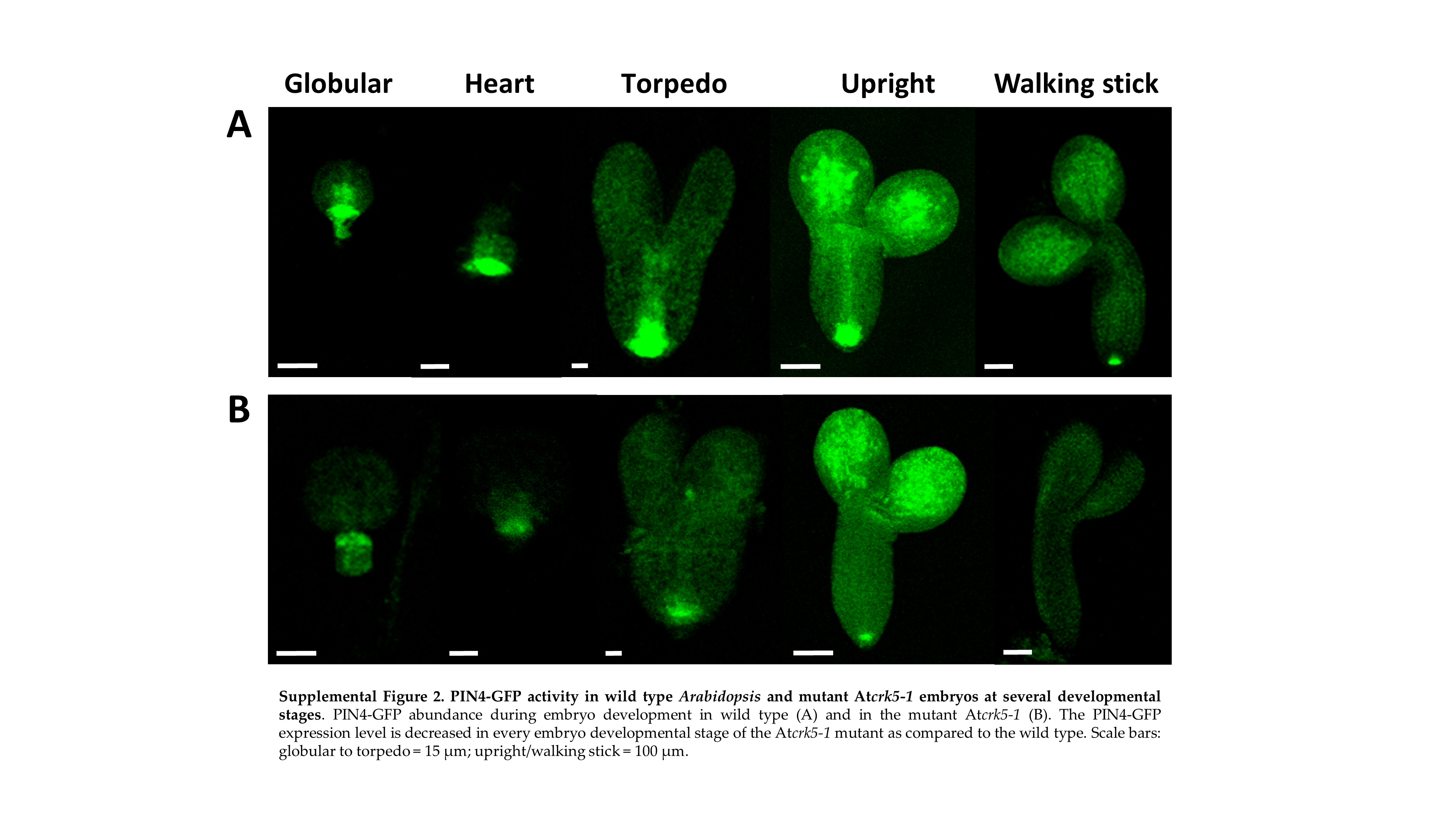

Supplement: Supplementary file 1 [file ijms-20-06120-s001.zip › Supplementary/Supplemental Figure 2.tif]

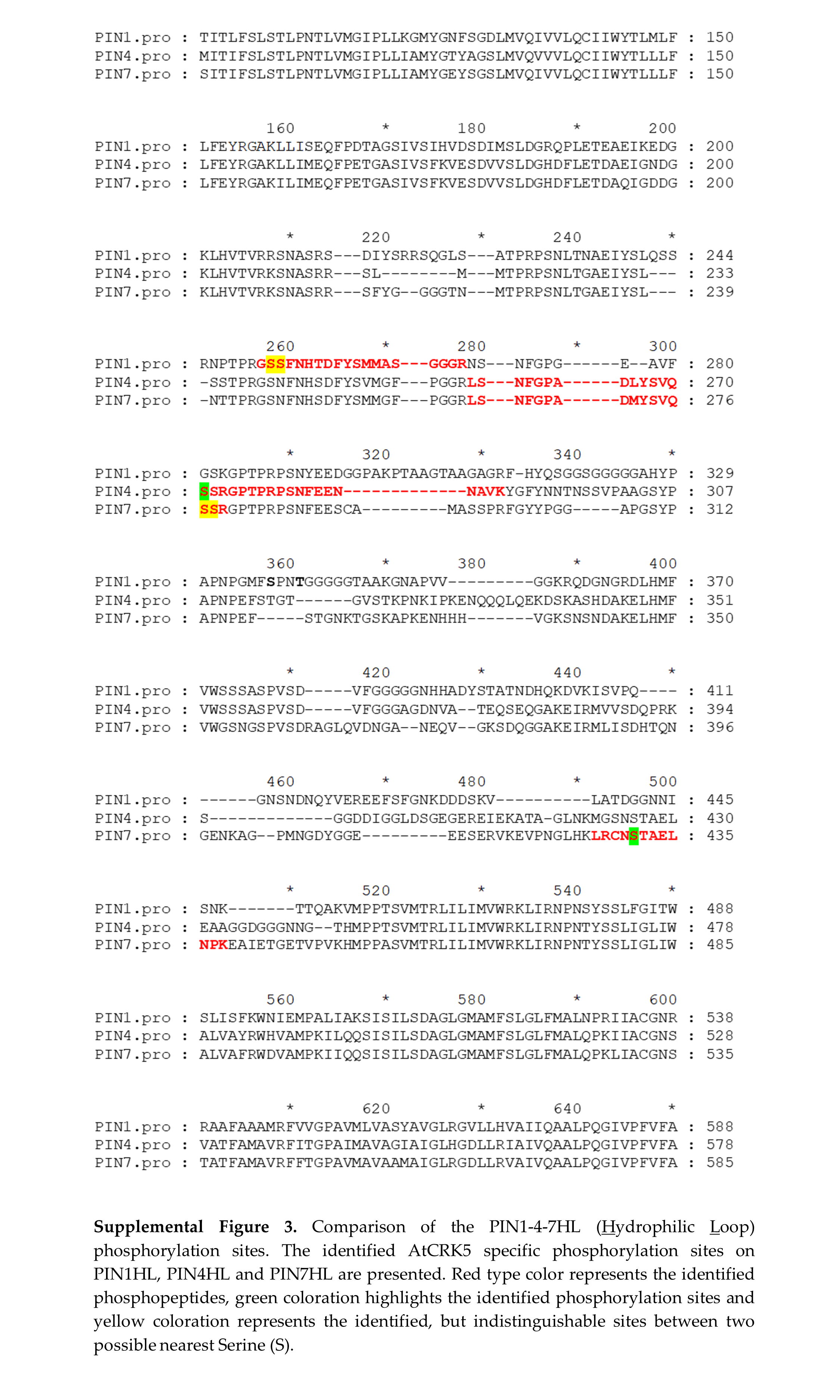

Supplement: Supplementary file 1 [file ijms-20-06120-s001.zip › Supplementary/Supplemental Figure 3.tif]

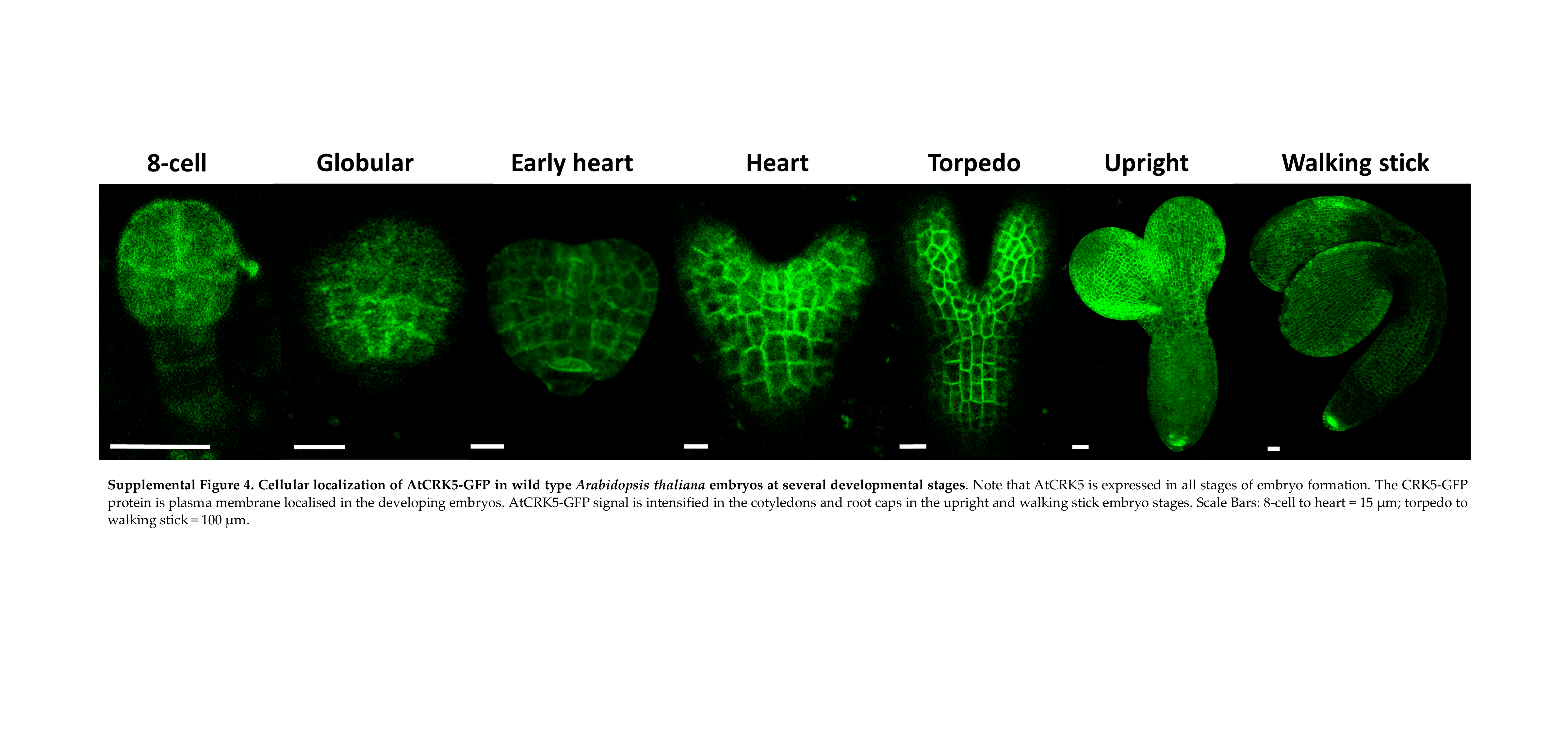

Supplement: Supplementary file 1 [file ijms-20-06120-s001.zip › Supplementary/Supplemental Figure 4.tif]

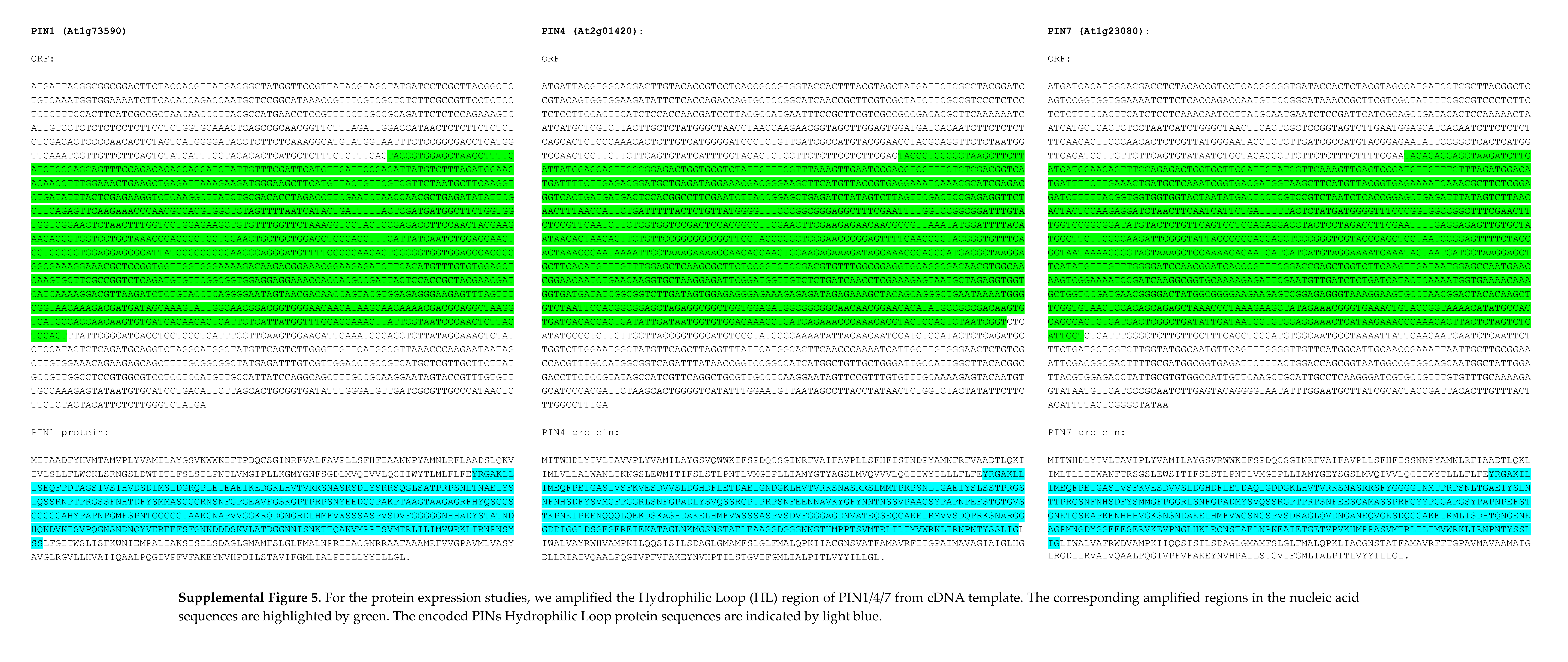

Supplement: Supplementary file 1 [file ijms-20-06120-s001.zip › Supplementary/Supplemental Figure 5.tif]
